# Immune Factor, TNFα, Disrupts Human Brain Organoid Development Similar to Schizophrenia—Schizophrenia Increases Developmental Vulnerability to TNFα

**DOI:** 10.3389/fncel.2020.00233

**Published:** 2020-08-28

**Authors:** Courtney A. Benson, Hana R. Powell, Michal Liput, Siddhartha Dinham, David A. Freedman, Tracey A. Ignatowski, Ewa K. Stachowiak, Michal K. Stachowiak

**Affiliations:** ^1^Department of Pathology and Anatomical Sciences, Jacobs School of Medicine and Biomedical Sciences, State University of New York at Buffalo, Buffalo, NY, United States; ^2^Department of Stem Cells Bioengineering, Mossakowski Medical Research Centre, Polish Academy of Sciences, Warsaw, Poland; ^3^Department of Biomedical Engineering, State University of New York at Buffalo, Buffalo, NY, United States

**Keywords:** organoids, tumor necrosis factor, schizophrenia, nuclear fibroblast growth factor receptor-1, neural progenitor cell, oligodendrocyte

## Abstract

Schizophrenia (SZ) is a neurodevelopmental genetic disorder in which maternal immune activation (MIA) and increased tumor necrosis factor-α (TNF-α) may contribute. Previous studies using iPSC-derived cerebral organoids and neuronal cells demonstrated developmental malformation and transcriptional dysregulations, including TNF receptors and their signaling genes, common to SZ patients with diverse genetic backgrounds. In the present study, we examined the significance of the common TNF receptor dysregulations by transiently exposing cerebral organoids from embryonic stem cells (ESC) and from representative control and SZ patient iPSCs to TNF. In control iPSC organoids, TNF produced malformations qualitatively similar in, but generally less pronounced than, the malformations of the SZ iPSC-derived organoids. TNF and SZ alone disrupted subcortical rosettes and dispersed proliferating Ki67^+^ neural progenitor cells (NPC) from the organoid ventricular zone (VZ) into the cortical zone (CZ). In the CZ, the absence of large ramified pan-Neu^+^ neurons coincided with loss of myelinated neurites despite increased cortical accumulation of O4^+^ oligodendrocytes. The number of calretinin^+^ interneurons increased; however, they lacked the preferential parallel orientation to the organoid surface. SZ and SZ+TNF affected fine cortical and subcortical organoid structure by replacing cells with extracellular matrix (ECM)-like fibers The SZ condition increased developmental vulnerability to TNF, leading to more pronounced changes in NPC, pan-Neu^+^ neurons, and interneurons. Both SZ- and TNF-induced malformations were associated with the loss of nuclear (n)FGFR1 form in the CZ and its upregulation in deep IZ regions, while in earlier studies blocking nFGFR1 reproduced cortical malformations observed in SZ. Computational analysis of ChiPseq and RNAseq datasets shows that nFGFR1 directly targets neurogenic, oligodendrogenic, cell migration, and ECM genes, and that the FGFR1-targeted TNF receptor and signaling genes are overexpressed in SZ NPC. Through these changes, the developing brain with the inherited SZ genome dysregulation may suffer increased vulnerability to TNF and thus, MIA.

## Introduction

Schizophrenia (SZ) is a neurodevelopmental psychotic disorder associated with fine changes in the brain cortical structure (Harrison, 1999). Both genetic factors, as well as physical and psychosocial environmental factors play a role in the development of SZ. The combined effect of genetics and environmental factors leads to abnormal circuit formation and consequently, aberrant neurotransmitter and synaptic function and ultimately abnormal behavior in SZ (Na et al., 2014; Estes and McAllister, 2016; Chuye et al., 2018). Evidence points to the primary hypothesis of convergence, in which genes and environmental factors converge and disrupt common developmental mechanism(s) leading to the emergence of SZ (Clarke et al., 2006; Schmitt et al., 2011).

A disrupted “feed forward and gate” signaling by integrative nuclear (n) FGFR1 signaling (INFS) during development acts as a common mechanism in genomic neurodevelopmental deprogramming in SZ patients with different genetic backgrounds (Narla et al., 2017). In cellular development, controlling signals propagate through diverse pathways to their end-point transcription factors. In parallel, these signals are fed forward by nFGFR1 directly to CREB binding protein (CBP), allowing genes to respond in a coordinated manner (Stachowiak and Stachowiak, 2016). Evidence indicates that in SZ, mutations of genes in diverse signaling pathways integrated by nFGFR1 disrupt the INFS function and thus neural development (Narla et al., 2018). Whether pathological environmental factors that contribute to SZ also affect nFGFR1, developmental function is unknown.

One environmental factor linked to the pathogenesis of SZ is infection during pregnancy and early childhood. Viral infection, particularly influenza, has been studied as a factor of SZ prevalence (Buka et al., 2001; Clarke et al., 2006; Patterson, 2009; Dean et al., 2013; Na et al., 2014). Maternal infection could alter immune status of the fetal brain and, along with genetic changes, significantly increase the risk of SZ. Elevated levels of the pro-inflammatory cytokine, tumor necrosis factor-α (TNF-α) have been reported in the placenta, amniotic fluid, and the brain of the fetus from mothers with influenza infection. Activated maternal TNF, and other cytokines, can invade the fetal CNS through various pathways, including disrupting the blood brain barrier (Buka et al., 2001; Clarke et al., 2006; Patterson, 2009; Dean et al., 2013; Na et al., 2014). TNF is one of the cytokines found to be significantly elevated in pregnant mothers that have been infected (Buka et al., 2001; Dean et al., 2013). TNF has been reported to affect neuronal growth, differentiation, synaptic scaling, and apoptosis in cultured cells and *in vivo* (Na et al., 2014).

Pregnant mice at mid-gestation infected with human influenza virus showed brain cortical layer and region-specific changes in the expression of the presynaptic marker, SNAP-25, iNOS, and Reelin (Patterson, 2009). Pyramidal cells were more densely packed, similar to what is observed in SZ (Patterson, 2009). Adult mice that were born to the infected mothers displayed an abnormality in neuronal migration to layer 2/3 in the cortex, which is similar to findings of downregulated DISC1 in SZ (Patterson, 2009) and in a recent SZ iPSC cerebral organoid study (Stachowiak et al., 2017). In mice, behavioral deficits were also observed in the offspring, including social interaction and open field and novel object exploration (Patterson, 2009), similar as in the FGFR1(TK-) transgenic mouse SZ model (Klejbor et al., 2006, 2009; Stachowiak et al., 2013). Increased levels of proinflammatory cytokines, including TNF, have consistently been reported in the blood and CSF of SZ patients during first and acute episodes (Buka et al., 2001; Clarke et al., 2006; Patterson, 2009; Dean et al., 2013; Na et al., 2014). Upregulated cytokine levels have been found in the adult brain of SZ patients, which could represent a permanent state of brain immune dysregulation (Buka et al., 2001; Clarke et al., 2006; Patterson, 2009). Increased TNF, inflammation and neuro-immune dysregulations have also been linked to autism spectrum disorders (Siniscalco et al., 2018).

In order to better understand the neurodevelopment of SZ, organoids, or “mini brains,” generated from patient iPSC, provided for the first time, an insight into early disease etiology, parallel to the *in utero* development of the fetal brain during disease development (Stachowiak et al., 2017). These cerebral organoids mimic and closely model human brain development by generating the cerebral cortex, ventral telencephalon, choroid plexus, and retinal identities, among other brain regions (Chuye et al., 2018). The production of organoids *in vitro* can be used for studies of migration, differentiation, basic early neurodevelopment, as well as disease development and progression. The cerebral organoids allowed for the first time to relate the broad INFS-linked transcriptional dysregulations found in SZ iPSC neural progenitor cells (NPCs; Narla et al., 2017) to development of the human brain malformations in SZ phenotype (Stachowiak et al., 2017). These genomic and tissue structural dysregulations were found in patients with different genomic backgrounds and may constitute a common developmental signature of the SZ phenotype (Chuye et al., 2018; Narla et al., 2018).

According to the “two-hit” theory of SZ, during gestation, genomic dysregulations (first hit) increase the fetus vulnerability to pathogenic factors, such as maternal immune activation (MIA) or hypoxia (second hit). This interaction between genetics and epigenetics could potentially affect histone acetylation and the methylation of DNA, and other gene regulatory mechanisms with lasting effects on neural development (Schmitt et al., 2011; Estes and McAllister, 2016). Despite having one of the two “hits,” there is a possibility of a child being born without the disease. However, having both “hits” seems to skew the statistics unfavorably against healthy neurodevelopment.

This study aimed to explore a potential interaction between genetic disease modeled in SZ iPSC cerebral organoids and in MIA modeled by organoid exposure to the cytokine TNF.

## Materials and Methods

### Cerebral Organoid Growth and TNF Exposure

Cerebral organoids derived from human (h) embryonic stem cells (ESC) and induced pluripotent stem cells (iPSCs) were grown according to our established protocol (Stachowiak et al., 2017) modified from the original studies of (Lancaster et al., 2013; Lancaster and Knoblich, 2014). The organoids had developed for 2 weeks (days 1–14) and at days 15–23 were transiently exposed to TNF (human recombinant TNFα, R&D Systems, Minneapolis, MN, USA). After an additional 2 weeks of recovery without TNF, the organoids were harvested on day 37. Fresh TNF was resupplied every 3 days along with medium change. Control organoids received fresh medium without TNF.

**Figure 1:**
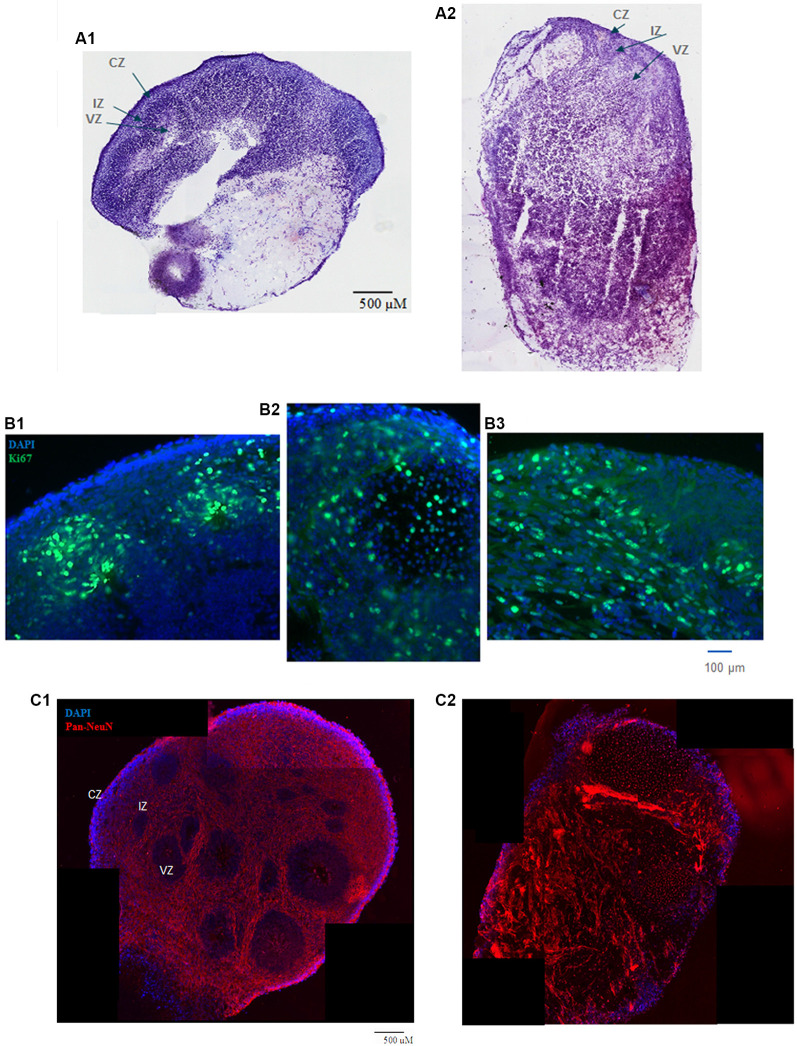
**(A)** HUES8s organoid at 5 weeks—tile scanning of NISSL staining. Representative images are shown of **(A1)** control, non-treated organoid, and **(A2)** Tumor Necrosis Factor-α (TNF-α) (1 ng/ml) treated organoid. **(B)** Rosette formation in HUES8 organoids at 5 weeks—immunostaining of Ki67+ proliferating cells (green immunostaining) and DAPI stained nuclei (blue). **(B1)** Control HUES8 organoids not exposed to TNF formed cortical rosettes with Ki67+ proliferative cells. **(B2)** In HUES8, organoids exposed to 1 ng/ml TNF and **(B3)** in HUES8 organoids exposed to 10 ng/ml TNF Ki67 positive cells were dispersed throughout the organoids. **(C)** Neuronal development in HUES8 organoids at 5 weeks, pan-Neu immunostaining—red, DAPI—blue, tile scanning. **(C1)** In control organoids, pan-Neu+ neurons were located throughout the organoids outside of the ventricular zones (VZ), in the intermediate zone (IZ) and were concentrated in the cortical zone (CZ). **(C2)** HUES8 organoids exposed to 1 ng/ml TNF exhibited disorganized pan-Neu neuronal development, with clusters of pan-Neu+ cells dispersed throughout the tissue, and concentrated in the scar-like region. Note fewer pan-Neu+ neurons in the CZ of TNF-exposed organoids.

ESC organoids were developed from the hESC line, HUES8-iCas9n as previously described (Stachowiak et al., 2017). The initial experiment was designed to determine the effective TNF concentration; 1 or 10 ng/ml of TNF was added to organoid cultures. These concentrations were based on earlier cell culture studies, which showed moderate effects of 20 ng/ml TNF on human neuronal viability (Talley et al., 1995), stimulation of gliogenesis and inhibition of neurogenesis by human brain NPC (Lan et al., 2012). Both 1 and 10 ng/ml concentrations affected the overall organoid development (Figure 1, Supplementary Figures S1, S2) and subsequently lower TNF concentrations (50 and 250 pg/ml) were used.

IPSC organoids were developed from one representative control (healthy individual) iPSC line BJ#1, and one SZ patient line, 1835 (Narla et al., 2017; Stachowiak et al., 2017). These representative lines were selected from four control and four SZ lines, which showed consistent differences in neuronal development (Brennand et al., 2015), gene expression including TNF receptor and signaling genes (Narla et al., 2017; Stachowiak et al., 2017), and in organoid corticogenesis between the apparently healthy and diverse SZ patients (Stachowiak et al., 2017). In the initial experiments leading to the current study TNF affected development of organoids of all these lines (CB, Ph.D. Thesis, 2017, University at Buffalo). The iPSC lines were originally obtained from Coriell Cell Repository, Camden New Jersey, NJ, USA. The iPSC organoids were exposed to 50 or 250 pg/ml TNF producing similar results. The four groups of iPSC organoids generated were control (C), control with TNF (C+TNF), schizophrenia (SZ), and schizophrenia with TNF (SZ+TNF) organoids. The organoids were grown and treated in separate batches including all treatment conditions. After freezing, the organoids were combined into common same treatment pools of which four organoids of the same treatment were randomly selected for analysis.

The organoid ventricular zone (VZ) is defined as cells surrounding rosette’s ventricle-like lumen, cortical zone (CZ) as the organoid cortex, and intermediate zone (IZ) as the intervening region. These zones were additionally characterized in Stachowiak et al. (2017) and in the present study as regions containing proliferating Ki67+ cells and non-differentiated stem-like radial glia adjacent to the rosettes’ lumen (VZ), migrating differentiating DCX+ neuroblasts (IZ), and βIII tubulin+ and differentiated pan-Neu+ neurons and calretinin+ interneuronsneurons (CZ). The intervening subplate region (between IZ and CZ) was earlier identified by anti-Reelin staining (Stachowiak et al., 2017).

### Immunohistochemistry

For each treatment group (C, C+TNF, SZ, SZ+TNF) four individual organoids were harvested, and sectioned at 30 μm. The immunohistochemistry was performed simultaneously on series of sections (as previously in Stachowiak et al., 2017), including of the four iPSC organoid groups with four organoids per group analyzed. The sections were incubated with the primary antibodies overnight at 4°C, rinsed 3× in 1× PBS and subsequently incubated with fluorescent secondary antibody in the dark for 2 h as described in (Stachowiak et al., 2017). Microscopic slides containing organoid sections were cover slipped with mounting medium (Fluoro Gel II with DAPI). The primary and secondary antibodies used are listed in Supplementary Table S1.

All the stains, except for FGFR1, were observed and imaged using a Zeiss Axio Imager Upright Fluorescence microscope equipped with Zen Blue 2.3 software. Images were taken at 10× and 20× magnification. For FGFR1 a Leica Upright Fluorescence microscope with LAS X software was used. The Ki67 images were analyzed using a MatLab script (Stachowiak et al., 2017). This script recognized individual cells with a red stain by selectively extracting information related to the red light channel. The individual red + cells in regions of interest (ROIs) were counted as a single event using catchment basin identification that used the regional maxima values to create distinct regions around individual events. Calretinin-expressing neurons were counted in ROIs and their longest axis marked by hand. A further MatLab-based calculation measured the angle of deviation of the axis of calretinin^+^ interneurons relative to overlaying organoid’s cortex using comprehensive program described in Stachowiak et al. (2017).

Pan-Neu and Olig4 Fluorescence Intensity Measurements were performed using Zen 2.0 Blue Imaging software in randomly selected ROIs within organoid regions (CZ and IZ) listed in the figure legends. As previously (Stachowiak et al., 2017), all images acquired were in the linear range of the camera. Images of sections from C, C+TNF, SZ, and SZ+TNF organoids were acquired under identical illumination conditions and identical camera gain, offset, and exposure times. Background images (outside the tissue sections) were taken and subtracted from the images of stained sections. The total area of each ROI image was recorded, as well as the number of threshold pixels and their integrated intensity. Each channel was converted into the 8-bit grayscale and the mean intensity was calculated.

Cells with nuclear FGFR1 (nFGFR1) immunostaining colocalized with the DAPI stain were counted in six CZ and six IZ ROIs using Visiopharm stereological software, as previously described (Stachowiak et al., 2017).

A recent study revealed the presence of neural crest cells in brain stem organoids (Eura et al., 2019). The presence of neural crest cells in the cerebral organoids was not investigated in the present study.

### Scanning Electron Microscopy (SEM)

SEM was performed along with fluorescent microscopy to verify the morphological differences in cortical surface in control and SZ organoids and following TNF exposure.

The 30 μm sections representing individual experimental conditions were fixed in 2% glutaraldehyde for 1.5 h at 4°C. The sections were dehydrated by successive immersion in gradients of ETOH (30, 50, 70, 90, and 100%) 15 min each, followed by dip in 100% hexamethyldisilanzane and air-drying. The dehydrated sections were mounted onto a SEM stage and processed for 10 min through a vacuum chamber, where a very thin carbon layer closely coated the entire surface of the sample to secure conductivity. Representative organoid sections from each of the four conditions were examined under a bright field microscope. The ROIs were identified and imaged on Hitachi S4000 (Field Emission SEM) with IXRF Energy-Dispersive X-Ray Spectrometer; University of Buffalo’s Instrument Center.

Images were taken at three different regions of each section with a special focus on the cortical and subcortical areas. At each region, images were taken at five increasing magnifications (500×, 1,500×, 2,500×, 5,000×, and 10,000×) without moving the stage. Circular ROIs were outlined, and numbers of cells/ROI were counted.

### RNAseq and ChiPseq Data Analysis

The RNAseq analysis was performed in our earlier study (Narla et al., 2017, 2018) using homogenous cultures of NPC differentiated from four control and four SZ patients’ iPSC lines. To induce neuronal committed cells, NPC were treated with 20 ng ml^−1^ BDNF (Peprotech), 20 ng ml^−1^ GDNF (Peprotech), 1 mM dibutyryl-cyclic AMP (Sigma), and 200 nM ascorbic acid (Sigma; Narla et al., 2017) for 2 days. The current analyses were performed on the RNAseq datasets (Narla et al., 2017) with the accession code: GSE92874. The ChiPseq data were generated from the NPC of two SZ and two control iPSC lines (Narla et al., 2017). The ChiPseq datasets analyzed in the present study can be accessed at GSE92873.

### Statistical Analyses

IBM SPSS was used to conduct one-way, two-way or three-way ANOVA followed by Fisher’s Least Significant Difference (LSD) *post hoc* analyses (Stachowiak et al., 2017). The details of sample sizes are listed in figure legends and fulfill the requirement of statistical significance.

## Results

### Human ESC Cerebral Organoids Recapitulate Cortical Development—Disruption by TNF

The initial experiments were performed on organoids generated from the human ESC line, HUES8, to test effects of different concentrations of TNF. Initially, the concentrations tested, 1 and 10 ng/ml, were based on cell culture studies, which showed their moderate effect on human neuronal viability (Talley et al., 1995), stimulation of gliogenesis, and inhibition of neurogenesis by human brain NPC (20 ng/ml; Lan et al., 2012).

HUES8 organoids were developed for 2 weeks in kinetic cultures and then incubated with 1 or 10 ng/ml of TNF for 9 days with fresh cytokine replenished every 3 days. After an additional 2 weeks of recovery in medium lacking TNF, organoids were harvested at day 37, the 5th week of the organoid development. In the control organoids, the culture medium lacking TNF was also exchanged every 3 days. At least six organoids were used per condition. At 5 weeks, the organoids reached 4–5 mm size and in the control TNF- lacking organoids, formation of multiple neurogenic rosettes (Figure 1A1) was observed. Similarly, as previously observed in ESC (H9) and iPSC organoids, there were three distinct regions, the VZ in the center of the rosettes, the surrounding IZ, and the peripheral CZ.

Incubation with TNF 1 ng/ml (Figure 1A2) or 10 ng/ml (not shown) disrupted the general organoid and rosette structures. The TNF exposed organoids stained with cresyl violet (Nissl staining) frequently displayed a scar-like tissue and their rosettes were irregular (Figure 1A). The scar-like regions contained displaced highly concentrated pan-Neu stained neurons (Figure 1C2) as well as GFAP stained glia (Supplementary Figure S1B). The latter may reflect a gliosis-like response to TNF observed *in vivo* (Livne-Bar et al., 2016; Galinsky et al., 2020).

At 5 weeks, the cortical rosettes of control HUES8 organoids contained Ki67^+^ proliferating NPC surrounding the central VZ lumen (Figure 1B1). Few Ki67^+^ cells were present in the IZ, and only single Ki67^+^ cells were occasionally found in the CZ. In 1 ng/ml (Figure 1B2) or 10 ng/ml (Figure 1B3), TNF-exposed organoids, Ki67^+^ proliferative cells were dispersed throughout the organoids, further illustrating the disruption of the rosette organization. Ki67^+^ cells spread throughout all zones including the CZ (Figures 1B2,B3) and were present in the scar-like damaged tissue (not shown). GFAP positive radial glia, the brain stem cells, which in control HUES8 organoids outlined the VZ (Supplementary Figure S1). The GFAP+ glia, were dispersed throughout the TNF-exposed organoids (Supplementary Figure S1B). Similar to the proliferating Ki67^+^ NPC, the GFAP-stained glia were found in the CZ and formed clusters in the scar-like regions of the TNF-exposed organoids.

Pan-Neu antibody cocktail, which stains mature neuron proteins of axons, dendrites, soma, and nuclei, showed neuronal development outside of the VZ. The pan-Neu^+^ neurons were concentrated in the CZ (Figure 1C1) and present in the subcortical area of the IZ around the VZ. TNF exposure markedly disorganized this neuronal pattern (Figure 1C2). Neurons appeared in dense clusters throughout the organoids, including in the scar-like tracts and were no longer outlining the rosettes and defining the CZ.

During brain development, a low level of apoptosis underlies cellular pruning. This process involves DNA binding by the effector caspase-3 (Prokhorova et al., 2018) and appears ongoing in the control cerebral organoids in which we found few apoptotic nuclei marked with anti-caspase 3 antibody (Supplementary Figure S2). Their occurrence increased in 1 and 10 ng/ml TNF-exposed HUES8 organoids, along with tissue damage and scar-like formation.

In summary, 1–10 ng/ml TNF exposure induced gross tissue damage throughout the organoids, formation of scar-like structures, increased apoptosis, disrupted neurogenic rosettes, and displaced neuronal formation.

### Dispersion of Ki67^+^ NPC in Schizophrenia iPSC Organoids and by TNF

The HUES8 organoids exposed to 1 or 10 ng/ml TNF accrued gross structural damage, along with fine cellular disruptions. Hence, in the subsequent iPSC experiments, we employed lower concentrations, 50 and 250 pg/ml of TNF to assess potential differences in the susceptibility of the control (C) and schizophrenia (SZ) iPSC organoids to TNF. The iPSC organoids were developed for 2 weeks and exposed for 9 days to TNF, and then maintained in TNF-free medium until day 37, as described for the HUES8 organoids.

At 37 days, we observed no gross differences of size, shape, or macroscopic deformities in the TNF exposed control (C+TNF) or schizophrenia (SZ+TNF) organoids compared to the C or SZ organoids that did not receive the cytokine (Supplementary Figure S3). Organoids receiving TNF at 250 pg/ml (Supplementary Figure S3) or 50 pg/ml (not shown) lacked the scar-like tissue that developed in organoids receiving 1 ng/ml and in 10 ng/ml TNF. Consistent with the previous study (Stachowiak et al., 2017), no gross structural differences were observed between the C and SZ organoids (Supplementary Figure S3).

**Figure 2:**
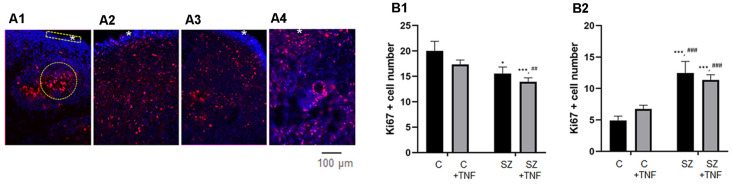
**(A)** Disorganized migration of proliferating cells in SZ and TNF exposed 5-week control (C) organoids; **(A1)**—C, **(A2)**—C+TNF, **(A3)**—SZ, **(A4)**—SZ+TNF. The images show organoids were immunostained for Ki67 (red) used to quantify cell densities in cortical regions of interest (ROIs; rectangle), *marks superficial cortical region. In organoids shown in **(A2–A4)**, note the dispersion of proliferating (Ki67+) cells outside the VZ into IZ and CZ. Examples of ROIs—circular—IZ, rectangular—CZ. **(B)** Changes in ki67+ cell densities. **(B1)** IZ—Density of Ki67+ cells in IZ was analyzed in circular ROIs [four sections per organoid of each of the four iPSC organoid groups (C, C+TNF, SZ, SZ+TNF), 1–3 ROIs per section]. Bars represent average ROI cell numbers per section. Two-way ANOVA compared—main effect of SZ, *F* = 10.44, *p* < 0.005; TNF, *F* = 3.07, *p* = 0.08. LSD *post hoc* analysis: *,***Different from C, *p* < 0.05, *p* < 0.0001; ^##,###^different from C+TNF. **(B2)** CZ—Density of Ki67+ cells in CZ was analyzed in rectangular ROIs (two to four sections per organoid of each group, three to seven ROI per section). Bars represent average ROI cell numbers per section. Two-way ANOVA—main effects of SZ, *F* = 40.2, *p* < 0.0001; no effect of TNF *F* = 0.139, *p* = 0.7; no significant interaction *F* = 2.34. *,***Different from C, *p* < 0.05, *p* < 0.0001; ^##,###^different from C+TNF.

Images of the C+TNF, SZ, and SZ+TNF organoid sections stained for Ki67^+^ and counterstained for DAPI nuclei revealed noticeable differences in the distribution of proliferating Ki67^+^ NPC (Figure 2A). In the C iPSC organoids, similar to C HUES8 organoids, the rosettes showed strong presence of Ki67^+^ proliferating NPC surrounded by blue (DAPI) rings of densely packed nuclei (Figure 2A1). The Ki67^+^ NPC were typically confined to the periventricular zone of the rosettes, fewer in the IZ, and only single in the superficial cortex. In the SZ and SZ+TNF organoids, the rosettes were less discernable as their Ki67+ cells appeared dispersed and spread into the CZ (Figures 2A3,A4). C+TNF organoids showed similar changes in the distribution of Ki67^+^ NPC as the SZ organoids, but these changes appeared less pronounced than in the SZ organoids (Figure 2A2).

To quantify changes in the distribution of the Ki67^+^ NPC, we counted cell densities within the CZ (5–6 cells deep in rectangular ROIs) and in the subcortical circular ROIs centered on the rosettes, including surrounding the IZ but excluding the cortical surface (examples on Figure 2A1).

In the IZ region, there was an overall significant effect of SZ, on the Ki67^+^ NPC density and nearly significant (*p* = 0.08) effect of TNF. The LSD *post hoc* analysis showed a significant reduction of Ki67^+^ cells in SZ and in SZ+TNF organoids compared to C. There was no significant additional loss of the NPC caused by TNF in the SZ organoids (Figure 2B1).

In the CZ, there was an overall significant effect of SZ (*p* < 0.0001), but no effect of TNF on the NPC density (Figure 2B2). The density of Ki67^+^ NPC increased over 2-fold in SZ and in SZ+TNF organoids. A similar trend observed in C+TNF organoids was not statistically significant.

### Schizophrenia and TNF Induce Changes in Cortical and Subcortical pan-Neu^+^ Neuronal Population

**Figure 3:**
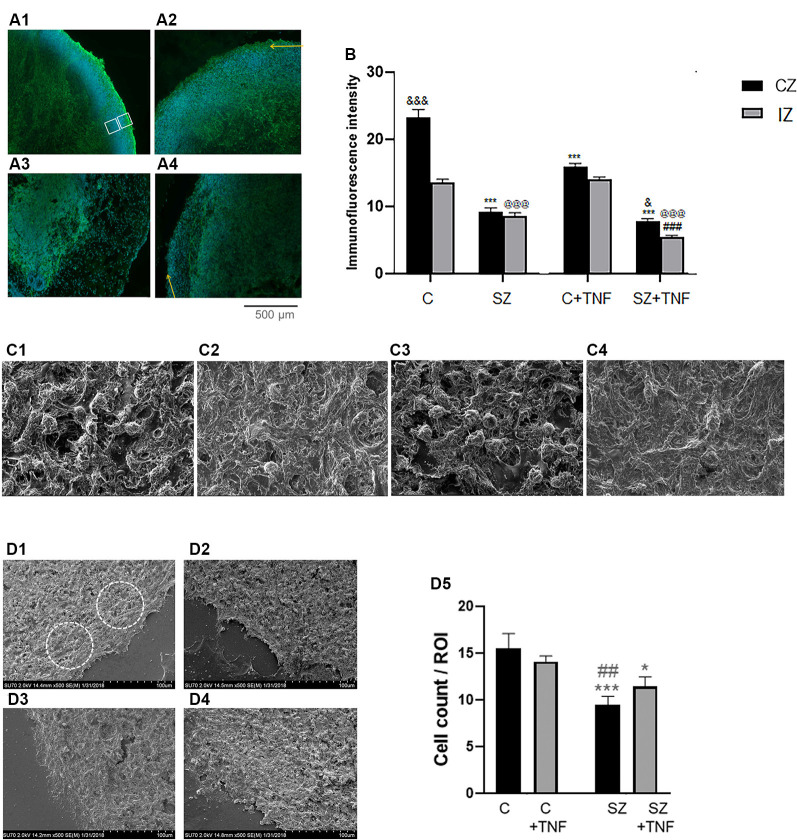
**(A)** Analysis of pan-Neu+ neuronal networks in 5-week iPSC organoids. Examples of the pan-Neu antibody stained images depicting the pan-Neu neuronal networks. In each image, pan-Neu immunofluorescence intensity was measured in five ROIs each in the cortical area (CZ), five in subcortical region of the IZ (examples on **A1**). Five additional ROIs placed outside the tissue were used for background subtraction (not shown), **(A1)**—C; **(A2)**—C + TNF, **(A3)**—SZ; **(A4)**—SZ + TNF. Yellow arrows point to cortical surface. **(B)** Quantitative analysis of pan-Neu intensity numbers in analyzed ROIs. Sixteen organoids, four from each condition and 10 sections from each organoid were analyzed for the pan-Neu immunofluorescence intensity as indicated on panel **(A)**; black bars—CZ, gray bars—subcortical IZ. Bars represent average ROI fluorescence intensity per section. Three-way ANOVA: significant main effects of SZ, *F* = 531.8 (*p* < 0.0001), TNF *F* = 55.33 (*p* < 0.0001), and organoid region *F* = 86.57 (*p* < 0.0001), as well as significant interactions (*p* < 0.0001), disease × organoid region, *F* = 29.22, and TNF × organoid region *F* = 15.91. In C organoids, the pan-Neu+ neuronal density was significantly higher in CZ than subcortical zone region of the IZ (*p* < 0.0001). *Post hoc* LSD: CZ, ***different from C (*p* < 0.0001), and different from SZ (*p* < 0.05); IZ, ^@@@^different from C (*p* < 0.0001), ^###^different from SZ (*p* < 0.0001). **(C)** Scanning electron microscopy (SEM) images of different organoid conditions: **(C1)**—C, **(C2)**—SZ, **(C3)**—C + TNF, **(C4)**—SZ + TNF; magnification 2,500×. Note reduced cell density and increased ECM fiber density in SZ and in SZ + TNF. **(D)** Cell densities were counted within circular ROIs (examples shown) in organoid images: **(D1)**—C, **(D2)**—SZ, **(D3)**—C + TNF and **(D4)**—SZ + TNF; magnification 500×. Panel **(D5)** shows results of cell counting. Two-way ANOVA: significant main effect of SZ, *F* = 15.73, *p* < 0.005. LSD: different from C (***p < 0.001; *p < 0.05); different from C + TNF (^##^*p* < 0.01).

The images of pan-Neu-stained organoid sections indicated a reduction in the neurons in cortical region CZ in all experimental conditions and less pronounced changes in the immediate underlying subcortical region of the IZ (Figures 3A1–A4). High density of pan-Neu+ cells and fibers precluded counting of the individual cells. To quantify changes in the pan-Neu^+^ neurons, we analyzed the intensity of Pan-Neu immunofluorescence in the cortical and subcortical IZ areas (Figures 3B1,B2). In the C organoids, the resulting intensity values indicated higher densities of the differentiated pan-Neu^+^ neurons in cortical region compared to the underlying subcortical IZ area (Figure 3B). These mature neurons formed a dense network of processes along with the networks in experimental conditions (Supplementary Figure S4).

Analysis of pan-Neu^+^ immunofluorescence intensity was performed by three-way ANOVA, which compared the effects of disease (C vs. SZ), TNF exposure (no TNF vs. TNF), and the organoid region: (CZ vs. subcortical IZ region; Figure 3B). ANOVA revealed a main effect of disease *p* < 0.001, whereby SZ conditions were associated with an overall decrease in neuronal staining intensity compared to non-disease organoids. There was a significant overall effect of TNF, *p* < 0.001, leading to decreased intensity of the pan-Neu staining. There were overall significant differences between the organoid regions, with the CZ being more intensely stained than the subcortical IZ region. There was a significant interaction between SZ and the organoid area, and between TNF and the organoid area, showing that the effects of the disease and of TNF were different in the CZ and IZ tissues.

*Post hoc* analysis using LSD verified that the intensity of the cortical region pan-Neu^+^ networks in C organoids was significantly greater than in C+TNF (*p* < 0.001), SZ (*p* < 0.001), or SZ+TNF (*p* < 0.001), indicating that both SZ and the addition of TNF disrupted the neuronal networks (Figure 3B). In contrast, in the subcortical region of IZ only SZ (*p* < 0.001) and SZ+TNF (*p* < 0.001) had depleted the pan-Neu^+^ neurons. TNF had an additional significant reducing effect on the IZ as well on the CZ neuronal networks in SZ + TNF compared to SZ. In control organoids TNF (C+TNF) had no significant effect on the pan-Neu^+^ network in the IZ, indicating that in SZ the pan-Neu^+^ neurons became vulnerable to TNF.

The severe cortical neuronal losses in SZ and SZ+TNF were reflected in the changes of the cortical structure as revealed by SEM. The SEM, magnification 1,500×, showed a number of changes in the cortical structure of the organoids exposed to TNF and in SZ organoids (Figures 3C1–C4). In SZ and in SZ+TNF organoids there were fewer cell bodies, more fibrous extracellular matrix-like (ECM) material and fewer unoccupied areas (lacking cell or fibers) than in C organoids (Figure 3C). Quantification of cell densities in identical size ROIs at 500× (Figures 3D1–D5) revealed a statistically significant reduction in the number of cells in the SZ and SZ+TNF organoids. A small reduction in C+TNF organoids did not attain statistical significance. Thus, the severe cortical neuronal loss in SZ and SZ+TNF is accompanied by a reduced density of the cortical cells, replaced by the ECM fibers, as detected by SEM.

### Distinct Effects of TNFα on Calretinin Expressing Interneurons in Control and SZ Organoids

**Figure 4:**
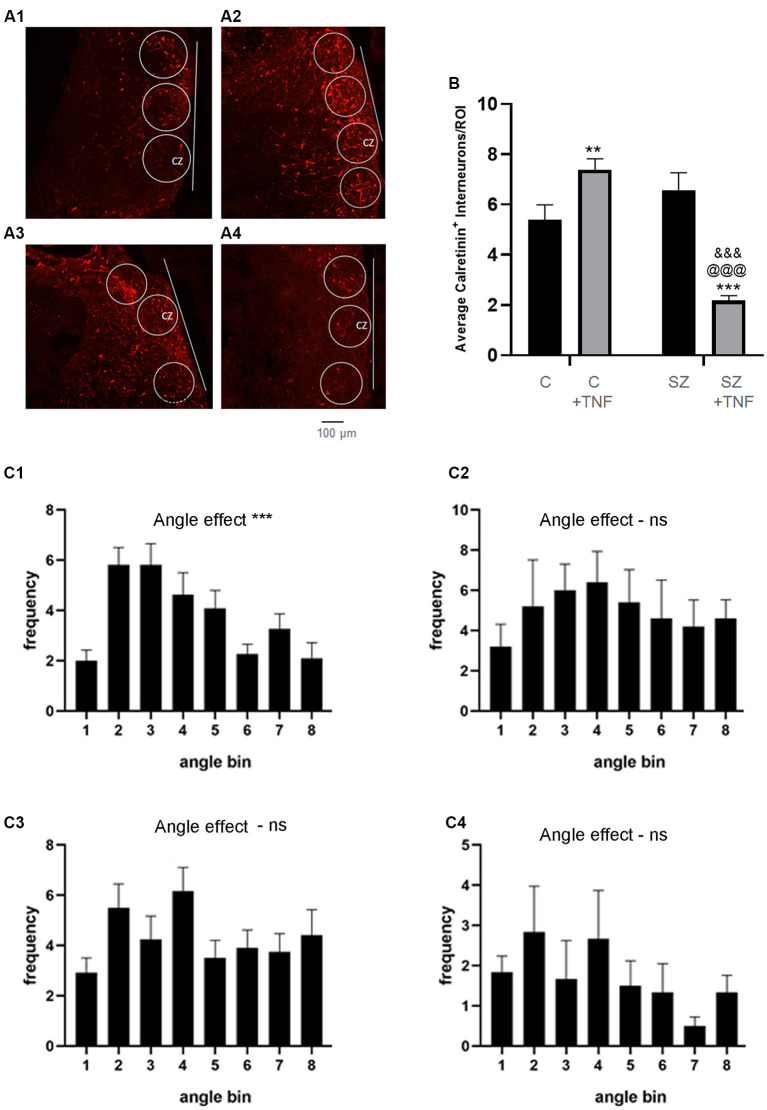
**(A)** Density and orientation of cortical calretinin interneurons. **(A)** Images of organoids immunostained for calretinin (red): **(A1)**—C, **(A2)**—C+TNF, **(A3)**—SZ and **(A4)**—SZ+TNF). ROIs were outlined in which densities and orientation (angles) of the Calretinin+ interneurons long axis relative to the organoid surface (indicated by line) were measured. Sixteen organoids were analyzed, four from each condition. A total 117 ROIs were placed across the CZ of the four conditions (27–30/condition). **(B)** The average cell density of the calretinin+ interneurons/ROI is shown. Two-way ANOVA analysis, showed a main significant effect of SZ *F*_(1,115)_ = 18.09, *p* < 0.001, main significant effect of TNF (*p* < 0.05) and a significant interaction between the disease and TNF exposure, *F*_(1,115)_ = 45.05, *p* < 0.0001. *Post hoc* LSD: **,***different from C (*p* < 0.0001, *p* < 0.005), ^@@@^different from C+TNF (*p* < 0.0001),^&&&^different from SZ (*p* < 0.0001). **(C)** Angles between the long axis of each calretinin+ cell and the cortical surface organoids were computed as described in the “Materials and Methods” section. Graph shows average frequency distribution of cells in ROIs in bins corresponding to the deviation angles from the cortical surface. Bin 1: 0–10°; 2: 10–20°; 3: 20–30°, etc. **(C1)**—C, c2—SZ, **(C3)**—C+TNF, **(C4)**—SZ+TNF. One-way ANOVA: main angle effect: **(C1)** *F*_(8,90)_ = 6.635, ***p < 0.0001, **(C2)** *F*_(8,36)_ = 0.97, *p* = 0.47, **(C3)** *F*_(7,88)_ = 1.65, **(C4)** *F*_(8,45)_ = 0.92, *p* = 0.5. ns = non-significant.

Calretinin is a protein expressed by a subpopulation of cortical GABAergic interneurons, which form intracortical connections of the cortical columns (Rogers, 1987), largely in the basal CZ and upper IZ (Figures 4A1–A4). We counted the numbers of calretinin^+^ interneurons within circular ROIs outlined in the cortical area (Figure 4A) and found a complex pattern of SZ and TNF-induced changes in calretinin^+^ cell density (Figures 4B, C1–C4). Two-way ANOVA analysis showed an overall (main) significant effect of SZ and a significant interaction between the disease and TNF exposure. Thus, SZ influenced the action of the cytokine. This was specified by *post hoc* LSD comparisons of the individual conditions, which revealed that TNF significantly increased the numbers of calretinin^+^ cells in C+TNF organoids compared to C (*p* < 0.001). A small increase observed in the SZ organoids was not significant; however, addition of TNF to the SZ organoids markedly depleted the calretinin^+^ interneurons compared to SZ (*p* < 0.001) and to C (*p* < 0.001) organoids (Figure 4B). Thus, while in the C organoids, TNF alone increased the number of calretinin^+^ cells, the combined actions of disease and TNF largely eliminated these interneurons.

Cortical interneurons connect cortical microcolumns by extending parallel largely to the cortical surface (Barinka et al., 2010). We measured the orientation angle of the individual cells’ long axes relative to the cortical surface using image J-based methodology. In C organoids, the greatest numbers of cells formed low angles (10–30°) with the cortical surface. This prevailing “near parallel” directionality of calretinin interneurons was maintained in C+TNF but was lost in SZ and in SZ+TNF organoids. ANOVA verified the significant effect of the angle factor on the cell frequency within different angle bins in C organoids and in C+TNF organoids and lack of such effects in SZ and in SZ+TNF organoids (Figure 4C).

### Changes in Neuronal Precursor Cells in Schizophrenia iPSC Organoids

The antibody against autism-linked transcription factor T-Box Brain 1 (TBR1), identifies developing neuroblasts of the subplate and cortical plate, which provide the first pioneer neurons of the developing cortical networks (Kolk et al., 2006). TBR1 is necessary for neuronal differentiation of NPC and is a potential master regulator in autism spectrum disorders (Chuang et al., 2015) and SZ (Stachowiak et al., 2017). At 5 weeks of the control iPSC organoid development, cells expressing moderate levels of TBR1 were distributed throughout the entire CZ (Supplementary Figure S5). As previously found, in the SZ iPSC organoids, the neuroblasts expressing high levels of TBR1 concentrated predominantly in the VZ proximal to the rosettes but were absent from the CZ region and were depleted in the cortical subplate IZ region. TNF exposure vastly depleted TBR1 cells in the CZ and IZ, as shown in the C+TNF organoids and TNF+SZ organoids, which displayed loss of TBR1 similar to SZ organoids that were not exposed to TNF (Supplementary Figure S5). Thus, impaired development of cortical neurons is associated with the absence of the cortical pioneer TBR1^+^ neuroblasts.

### SZ and TNF Influence Distribution of Oligodendrocytes in Developing Organoids

The O4 antibody stains late oligodendrocyte progenitors, by reacting with a sulfated glycolipid antigen (Proligodendrocyte Antigen, POA). It also reacts with oligodendrocytes that have entered terminal differentiation, in which case it reacts with sulfated galactosylcerebroside, sulfatide (Ohnishi et al., 2013).

**Figure 5:**
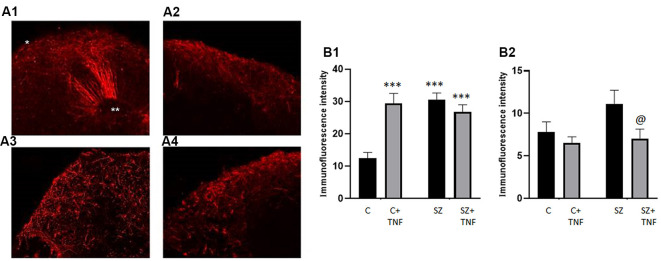
Distribution of O4 Oligodendrocytes is affected in SZ and by TNFα. Panel **(A)** shows the florescent microscopy images of O4 antibody stained organoids (red). **(A1)**—C, **(A2)**—C+TNF, **(A3)**—SZ, **(A4)**—SZ+TNF. The radial scaffolding of migrating O4 cells emanating from ventricular rosettes towards the cortex in the C organoids **(A1)** was largely lost in C+TNF, SZ, and SZ+TNF organoids **(A2–A4)**. In TNF-treated and in SZ organoids, conditions O4 oligodendrocytes were largely restricted to the cortical region. *CZ, **VZ. **(B)** Changes in O4+ immunofluorescence intensity induced by TNF and in SZ. A total of 16 organoids were analyzed, four from each condition; CZ—cortical region, IZ—subcortical region. A total of 128 ROIs were analyzed, with eight ROIs/image, four in CZ—cortical region, four in IZ—subcortical region. In each image, four additional ROIs were placed outside and use for background subtraction, but were not part of the total count. **(B1)**—CZ, two-way ANOVA: significant main effect of SZ *F* = 11.04, *p* < 0.05, TNF *F* = 8.088, *p* < 0.05, SZ × TNF interaction *F* = 19.81, *p* < 0.0001. **(B2)**—IZ, two-way ANOVA: significant effect of TNF *F* = 5.054, *p* < 0.05. LSD: ***different from C (*p* < 0.0001); ^@^different from SZ (*p* < 0.05). In addition a three-way ANOVA of combined CZ and IZ showed significant main effects of SZ *F* = 13.67 (*p* = 0.0003), and organoid region *F* = 163.4 (*p* < 0.0001) as well as significant interactions, disease × TNF *F* = 20.1 (*p* < 0.0001), disease × organoid region *F* = 4.915 (*p* = 0.0285), TNF × organoid region *F* = 12.65 (*p* = 0.0005) and disease × TNF × organoid region *F* = 11.74 (*p* = 0.0008).

In C organoids, the O4^+^ oligodendrocytic cells followed radial patterns extending from the VZ of subcortical rosettes, through the IZ and to the CZ (Figures 5A1–A4). However, in all experimental conditions, C+TNF, SZ, and in SZ+TNF this radial pattern were lost. The VZ regions were devoid of O4 cells, while the majority appeared restricted into the cortical region. To quantify these changes, the intensity of the O4 immunofluorescence was measured in two areas: CZ and subcortical IZ, and the results are shown on Figures 5B1,B2, respectively. In the CZ, two-way ANOVA showed significant main effects of SZ, TNF, and their interaction. *Post hoc* LSD test verified significant increases in O4 densities in C+TNF, SZ, and SZ+TNF compared to C. In the IZ, we found a significant main effect only of TNF. The O4 cell density was reduced by TNF only in SZ (SZ+TNF < SZ).

In addition, three-way ANOVA of the combined CZ and IZ results showed significant main effects of SZ, TNF, organoid region (IZ vs. CZ) as well as significant interactions of SZ × organoid region and TNF × organoid region (in contrast, the pan-Neu+ neuronal density was significantly higher in CZ than in the subcortical zone region of the IZ (Figure 3B). Thus, the effects of TNF differed between the C and SZ organoids and between the IZ and CZ. Interestingly, the effects of SZ and TNF on the CZ O4 oligodendrocytic cells (Figure 5B1) and pan-Neu neurons (Figure 3B), were opposite, resulting in accumulation of oligodendrocytes and loss of neurons from the organoid cortical area.

To assess if the changes in the distribution of O4 oligodendrocytes were reflected in neuronal myelination, we stained the organoid sections for myelin basic protein (MBP) along with pan-Neu. In the C organoids, we observed closely co-localized pan-Neu^+^ and MBP^+^ staining indicating the presence of myelinated neurons in the CZ and IZ (Supplementary Figure S6A). In SZ organoids, we observed fewer myelinated neurons in the CZ and IZ (Supplementary Figure S6A) indicating lack of myelination. In C+TNF organoids, pan-Neu and MBP co-staining persisted in the CZ similar to C organoids, thus, the generation of myelinated fibers in the CZ was unchanged. However, consistent with loss of subcortical and deep IZ O4 oligodendrocytes, the myelin staining in deep areas of the C+TNF organoids was markedly reduced (Supplementary Figure S6A). In the SZ, as well as the SZ+TNF organoids, the CZ had reduced neuronal staining and lacked myelinated fibers while the IZ contained unmyelinated neurons (Supplementary Figure S6A).

### Expression of nFGFR1 Protein Is Reduced in the Cortex of the SZ and TNF Exposed Organoids

The nuclear form of FGFR1 (nFGFR1) is a central signaling protein of the Integrative Nuclear FGFR1 Signaling (INFS). nFGFR1 activates coordinated gene programs, which promote neuronal development and inhibits the genes involved in oligodendrogenesis (Stachowiak and Stachowiak, 2016; Narla et al., 2017). In contrast, cytoplasmic/plasma membrane associated FGFR1 is expressed in proliferating non-differentiated cells, including NPC, and stimulates cell proliferation (Stachowiak and Stachowiak, 2016).

**Figure 6:**
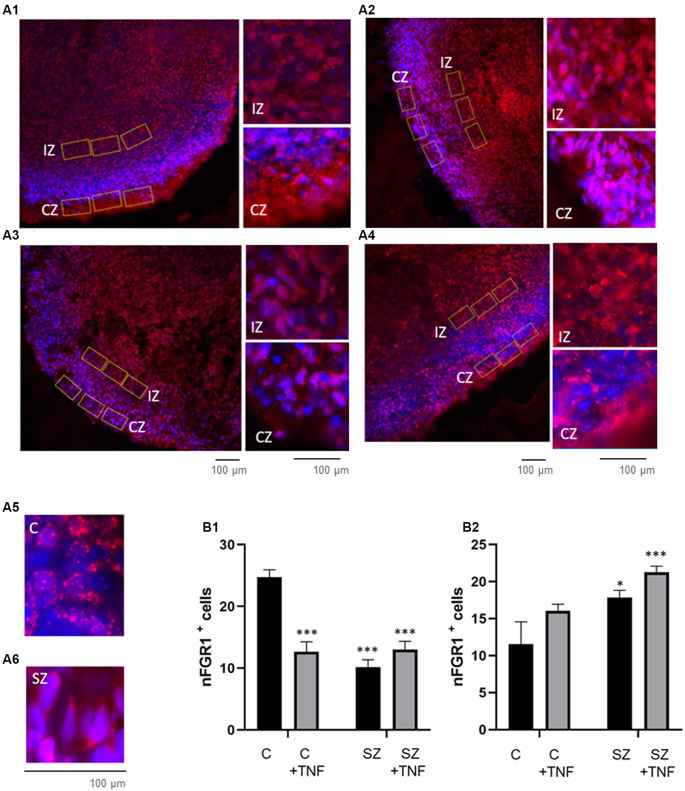
Increases in nFGFR1 expression in subcortical cells and loss of nFGFR1 expression in cortical cells in SZ organoids and induced by TNF. **(A)** Immunostaining of FGFR1 (red) and co-staining with DAPI (blue). Organoids: **(A1)**—C, **(A2)**—C+TNF, **(A3)**—SZ, **(A4)**—SZ+TNF. In C organoids the nFGFR1 was highly expressed in the CZ cells and less expressed in the IZ cells. This pattern was reversed in SZ and TNF conditions, where nFGFR1 was depleted in the CZ and more highly concentrated in the IZ. Examples of different subcellular localization of FGFR1 staining: nuclear in C **(A5)** and cytoplasmic in C **(A6)**, CZ organoid area. Red/pink speckles on a blue DAPI background represent nFGFR1, while cytoplasmic FGFR1 forms red cytoplasmic staining surrounding the blue DAPI stained nuclei. For each image, three of the same ROIs were placed in the CZ and three in the IZ. **(B)** Sixteen organoids, four from each condition and four images from each organoid were analyzed for the number of cells with nFGFR1 (colocalized FGFR1 and DAPI stains). For each image, the total number of cells with nFGFR1were counted in three ROIs in CZ **(B1)** and in three ROIs in the IZ **(B2)**. Bars represent average total number of nFGFR1+ cells per image. **(B1)**—CZ—two-way ANOVA main effects of SZ *F* = 27.81 (*p* < 0.0001) and TNF *F* = 11.84 (*p* < 0.005); significant interaction between the SZ and TNF *F* = 30.48 (*p* < 0.0001). **(B2)**—IZ, main effect of SZ *F* = 10.71 (*p* < 0.005), TNF *F* = 0.641 (*p* < 0.05). LSD: *,***different from C *p* < 0.05, *p* < 0.0001.

Five-week-old organoids were imaged for anti-FGFR1 immunofluorescence along with DAPI (Figures 6A1–A6) to identify its subcellular distribution. In C organoids, strong nFGFR1 staining was observed in the CZ where it colocalized with the DAPI DNA stain, thus representing nFGFR1. nFGFR1 staining in the IZ was weaker, with fewer cells showing FGFR1/DAPI colocalized stains, while some cells displayed cytoplasmic FGFR1. In SZ organoids, as shown previously (Stachowiak et al., 2017) the nuclear presence of FGFR1 in the CZ was reduced, while it appeared increased in the IZ. This altered pattern of nFGFR1 expression was observed also in C+TNF and in SZ+TNF organoids (Figure 6A).

To quantify these changes, we counted nFGFR1+ cells, where nFGFR1 immunostaining co-localized with the nuclear DAPI stain. Counting was performed in rectangular ROIs within the CZ and IZ regions as illustrated in Figures 6A1–A4. The effects of disease (C vs. SZ) and TNF (no TNF vs. TNF) on the number of nFGFR1 positive cells were compared using a two-way ANOVA (Figures 6B1,B2). In the CZ, significant main effects of both SZ (*p* < 0.000) and TNF (*p* < 0.01) were found. There was also a significant interaction between the disease and TNF (*p* < 0.0001), thus, SZ affected the organoid response to TNF. *Post hoc* analysis verified that both TNF and SZ depleted cortical nFGFR1^+^ cells and that their effects were not additive. In the IZ, both SZ and TNF had main significant effects (*p* < 0.005 and *p* < 0.05, respectively), but no interactions were found. *Post hoc* comparisons showed a significant increase in the numbers of FGFR1^+^ cells in SZ and an additional increase in SZ+TNF organoids. The increase observed in C+TNF did not reach statistical significance. These results indicated an overactivity of INFS in the IZ cells of SZ and SZ+TNF organoids and a loss of INFS activity in the CZ cells of all disrupted conditions.

### nFGFR1 Regulates Genes of Neuronal and Oligodendrocytic Differentiation, Cell Migration, ECM, and TNF Signaling

The study by Narla et al. (2017, 2018) identified genes dysregulated in SZ NPC as belonging to the general Gene Ontology categories of neuronal development (overexpressed genes) and oligodendrogenesis (underexpressed genes). The exemplary genes of these two categories were found to be affected by nFGFR1: stimulated (neuronal genes: TH, DISC1, Wnt7B, NeuroD) and inhibited (oligodendrocytic genes: Olig 2, Olig 1; Narla et al., 2017). We recently reported that dysregulation of nFGFR1 in the developing NPC progeny directly affected genes representing additional ontogenic processes, including cell migration and ECM, all of which were disrupted in SZ and in organoids exposed to TNF (Stachowiak et al., 2017). Many of the SZ dysregulated genes had promoters targeted by nFGFR1 (Narla et al., 2017; ChiPseq datasets GSE92873) and were directly affected by the changes of nFGFR1 signaling (Narla et al., 2017). Here, we analyzed nFGFR1 binding to the promoters of the exemplary genes involved in generation and function of neurons, oligodendrocytes, cell migration, and ECM using the NCBI Genome Browser and ChIP-seq datasets GSE92873. Genome browser illustrations of nFGFR1 binding to its promoters are shown in Supplementary Figures S7–S10. The analysis of our FGFR1 ChiPseq data revealed that nFGFR1 binds directly to the promoters of genes that underwrite neuronal development: WNT7B, Disc1, Neurofilament NEFL, NEFM and NEFL (Supplementary Figure S7); to the promoters of oligodendrogenic genes: Olig 2 and Sox9 (Supplementary Figure S8), ECM genes: ITGB3, NCOA3, POMT1, ANXA2, ADAMTS14 (Supplementary Figure S9) and cell migration genes: Ema4G, MSN, Syne2 (Supplementary Figure S10). Thus, changes in nFGFR1 expression in the IZ and CZ may provide a direct mechanism through which the ontogenic processes became dysregulated in the SZ and TNF exposed organoids.

Mechanisms by which SZ could affect the responses of NPC in organoids to TNF were explored by comparing the expression of TNF receptors and signaling genes in the RNAseq datasets (GSE92874) from control and SZ NPC deposited along with (Narla et al., 2017). The genes upregulated in SZ NPC included TNFRSF1B encoding TNFR2, TNFRSF10B (so called “Death Receptor” 5.5 log2), and genes for proteins that transduce TNF receptor signals, PAK1 (3.3 log 2), and PAK7 (2.2 log2; P21 protein activated kinases). These genes were affected by transfected constitutively active and/or dominant negative nuclear FGFR1 FGFR1(SP-/NLS) (Stachowiak et al., 2017; RNAseq datasets GSE103307) that have promoters targeted by nFGFR1 (Supplementary Figure S11). These findings indicate that overactive TNF signaling in developing SZ NPC may be instated by overactive INFS and affect the responses of the SZ organoids to TNF.

## Discussion

SZ is a polygenetic disorder in which environmental influences, such as MIA, are thought to play an enabling or exacerbating role. With over 300 genes linked to SZ, it has been proposed that these genes converge on a common developmental mechanism, the disruption of which increases the risk of the disease (Cannon and Keller, 2006; Chuye et al., 2018). In support of this model, recent studies demonstrated a common dysregulated transcriptome of over 1,300 genes in developing neurons shared by SZ patients with different SZ-linked mutations (Narla et al., 2017). The pan-ontogenic INFS mechanism, in which SZ-linked genes converge, is dysregulated in all such patients. Over 80% of dysregulated genes are targeted by nFGFR1, and the changes in nFGFR1 signaling disrupt the ontogenic gene programs that lead to developmental malformations as found in SZ NPC and in cerebral organoids (Narla et al., 2017).

Our present study shows that TNF, similar to SZ, alters nFGFR1 signaling, NPC, and neurons in developing cerebral organoids that together negatively affects brain development modeled in organoids. The dysregulation of INFS instigated by SZ genetic changes is reproduced by TNF, and thus, may serve as the converging point in the genetic-environmental etiology of SZ and related disorders. In addition, exposure of the SZ patient’s cerebral organoids to TNF exacerbates a number of cellular malformations and thus, may contribute to the development of brain pathology in SZ.

### TNF in Schizophrenia

One environmental factor, which significantly increases the odds of SZ in at-risk individuals, is MIA. When an expectant mother undergoes an infection-induced immune activation, the increased levels of the maternal cytokines can be transmitted across the placenta and potentially affect neurodevelopment of the human fetus (Toder et al., 2003; Estes and McAllister, 2016). TNF is a cytokine, whose effects can be either protective or harmful, dependent on its source, concentration and the existing brain environment (Perry et al., 2002). In the absence of MIA, endogenous brain TNF acts as a neuromodulator that supports normal brain development. However, when the brain becomes inflamed, the additional TNF produced by microglia and macrophages becomes neurotoxic (Perry et al., 2002). In the rodent brain, ischemia-induced inflammation switches the TNF function from neuroprotective to neurotoxic (Perry et al., 2002). While there is no evidence that fetal brain inflammation occurs in SZ, studies have shown elevated serum TNF levels that correlate with the incidence of SZ (Lee et al., 2017). Patient studies revealed elevated TNF blood levels, indicating an overall increase in TNF production (Ajami et al., 2014; Zhu et al., 2018). The risk of SZ increases as the immune cytokine levels are increased during pregnancy (Canetta and Brown, 2012). Specifically, the mothers of offspring who later went on to develop SZ had significantly elevated levels of TNF and interleukin-8 in the second and early third trimester, relative to the mothers of offspring who did not develop SZ (Canetta and Brown, 2012).

Furthermore, changes in brain chemistry and structure caused by broad dysregulation of genes, including TNF signaling proteins shown in our investigation, could alter the response of the developing brain stem cells, neurons, and oligodendrocytes to TNF (Narla et al., 2017; Stachowiak et al., 2017). During MIA, the increased levels of the maternal produced TNF may become transmitted across the placenta and act on the developing brain as a harmful neurotoxin (Toder et al., 2003; Estes and McAllister, 2016).

TNF pg/ml serum concentrations have been reported in human studies (Díez et al., 2002). The ED_50_ of TNF is ≤1 ng/ml and similar concentrations are frequently used in cell culture studies (Talley et al., 1995; Lan et al., 2012). The 1 ng/ml and 10 ng/ml TNF doses used initially in our study resulted in gross morphological malformations, as well as cellular disruptions in HUES8 cerebral organoids. The fine cellular malformations were observed with 200-fold lower concentrations of TNF, indicating that relatively small concentrations of TNF carry a significant risk of affecting brain development and have increased pathogenicity in the SZ organoids. Hence, cerebral organoids emerge as an effective model to study the neurotoxic effects of cytokines and other brain-affecting factors.

### TNF and SZ Induced Malformations in Developing Brain and Interplay of TNF and SZ

We previously described a time-dependent generation and development of organoids, recapitulating the inside-out pattern of human cortical development (Stachowiak et al., 2017), similar to other laboratories that pioneered this new technology (Lancaster et al., 2013; Lancaster and Knoblich, 2014). The VZ in the center of the organoid rosettes contained GFAP^+^ radial glia and proliferating NPC, the IZ contained migrating doublecortin^+^ neuroblasts and immature βIII-tubulin^+^ neurons, and the CZ incorporated pioneer neurons (TBR1^+^) and built cortical-like layers of the βIII-tubulin^+^, pan-Neu^+^ neurons, and calretinin^+^ interneurons (Stachowiak et al., 2017). These cellular structures, zones, and their development were consistently reproduced among organoids generated from multiple hESC lines and control iPSC lines but were noticeably disrupted in SZ iPSC organoids. The same structural and cellular disruptions were established in the organoids of patients carrying different SZ-linked mutations (Stachowiak et al., 2017), similar to the present results, indicating that they are general features of the developing SZ brain. We show that with addition of TNF to either ESC or iPSC organoids, the C+TNF and SZ organoids were similar on many accounts, but different from the C organoids. One of the most striking features of the SZ organoids and of the TNF exposed ESC and iPSC organoids was the disruption of their developmental strata. Specifically, we observed a movement of proliferating Ki67^+^ NPC from the rosettes in the VZ into the CZ. In the organoids developed from either hESC or control iPSC, the proliferating cells were restricted to two to three layers surrounding the lumen of the VZ rosettes. Relatively few cells that migrated to the IZ remained in a proliferative state, and essentially, no such cells were found in the CZ. In contrast, in C+TNF and SZ+TNF, similar as in the SZ organoids, much of the Ki67^+^ cells were dispersed out from the VZ and accumulated in the CZ. Thus, the endogenous disease and the introduction of TNF reduced the number of active proliferating NPC in the neurogenic rosettes, verified by cell counting, and caused their dispersion into other organoid zones. The disorganization of the neurogenic rosettes by TNF was also revealed by dispersion of GFAP^+^ radial glia, neural stem cells, which similar to Ki67^+^ NPC, appeared outside the rosettes across the organoid interior. Hence, we conclude that both the immune cytokine, TNF, and the diseased brain state, caused rampant migration of the proliferating cells outside the normal confines of the VZ. Such dispersion of the NPC was accompanied by an abnormal formation of neuronal foci in deep subcortical regions of the organoids and excessive numbers of cortical oligodendrocytes.

Despite expanded subcortical neurogenesis and cortical oligodendrogenesis, there were fewer mature neurons expressing high levels of pan-Neu neuronal markers in the cortex of SZ organoids (see also Stachowiak et al., 2017) and in TNF-exposed organoids, both C and SZ. This was shown by a significant reduction in cortical pan-Neu intensity (Figure 3B) and was corroborated by visibly diminished pan-Neu fibers in the SZ and in the organoids exposed to TNF (Supplementary Figure S2). The loss of cortical neurons was indicated as well as by fewer cortical cells as shown by SEM in Figure 3D.

In C+TNF, SZ, and SZ+TNF organoids, the specific depletion of the neurons in the cortex, but not in the IZ, where formation of abnormal neuronal foci was observed, was accompanied by the reduced cortical migration of TBR1^+^ neuroblasts and their accumulation in the IZ (Supplementary Figure S3). The present study verified our earlier observations and indicate an abnormal subcortical arrest of pioneer TBR1 neurons (Stachowiak et al., 2017) and lack of their cortical migration in the SZ organoids (Stachowiak et al., 2017). In SZ organoids, the reduced cortico-petal migration of TBR1^+^ neuroblasts and immature neurons correlated with the diminished deposition of reelin in the organoid cortex (Stachowiak et al., 2017). Further studies will be carried out to determine if an impaired reelin guidance may underlie the reduced formation of the cortical neuronal layers in TNF organoids, similar to SZ. Together, these findings illuminated an abnormal subcortical neuronogenesis in SZ and by TNF and were consistent with the premature neuronal generation predicted from the transcriptome studies of the NPC derived from SZ iPSC (Narla et al., 2017, 2018).

In SZ organoids, as shown in Figure 3B, and in the previous study (10), there was a reduced formation of the pan-Neu mature neurons in the top and basal regions of the cortex. In contrast, in control organoids, TNF depleted only superficial cortical neurons (Figure 3B), similarly as observed in mouse studies of immune activation during fetal neurodevelopment (Soumiya et al., 2011). However, in SZ organoids, TNF augmented the overall cortical neuronal loss, especially in the basal cortex, pointing to an increased vulnerability of the disease-affected cortex to this cytokine. These findings reinforce the notion that SZ and exogenous TNF have a cumulative negative effect on human cortical development, as modeled by cerebral organoids. The interaction of the SZ disease and TNF was also evident in the changes in calretinin^+^ interneurons, the density of which was not affected in SZ organoids, increased in C+TNF organoids, but was markedly depleted in SZ+TNF organoids. Although pyramidal neurons comprise more than 90% of cortical neurons and they project to target regions, local inhibitory interneurons shape pyramidal cell firing and their timing. The altered relationship between excitatory neurons of the vertical microcortical columns and the connecting horizontal GABAergic interneurons (of which calretinin positive neurons are a subtype) has been suggested in relation to the development of SZ in humans (Reynolds et al., 2002; Murray et al., 2014). Specifically the reduced function of inhibitory interneurons has been linked to deprograming of SZ patients cortical circuits (Sullivan and O’Donnell, 2012). Our study suggests that TNF induced a decrease in interneuron numbers in SZ organoid cortex, and their altered directionality in SZ and following TNF exposure may affect cortical connections and communication in SZ following MIA. Whether the disorientation of calretinin interneurons (Figure 4) could be a consequence of the disorganized proliferating progenitor cells (Figure 2) is currently unknown.

### SZ and TNF Have Opposite Effects on Oligodendrocytic Development in the Cortex (Increases) and Subcortical Zone (Decreases) and Affect Neuronal Myelination

A striking feature of both the SZ and TNF exposed organoids were marked increases in the O4^+^ cells within the cortical layers. The increased O4 intensity may reflect increased expression of O4 by individual cortical cells, potentially a result of an accelerated differentiation of cortical oligodendrocyte progenitors (OPC) to O4 oligodendrocytes. Alternatively or in addition to the proposed above, the higher O4 intensity could reflect increased numbers of the developed oligodendrocytes. The latter is consistent with increased presence of proliferating Ki67+ cells, some of which may represent OPCs. The presence of oligodendrocytic cells in human cerebral organoids was shown in this study and consistent with this finding, we observed the pan-Neu^+^ and MBP co-stained axons of the myelinated neurons. In 5-week organoids, as the dense neuronal network formed, the occurrence of the myelinated fibers was noticed within the CZ and IZ. The development of myelinated neurons was conspicuously diminished in the SZ organoids, in C+TNF organoids and no additional effect of TNF could be noted in SZ+TNF. Despite the increased presence of O4^+^ oligodendrocytic cells in cerebral organoids, the diminished developing population of pan-Neu^+^ neurons remained largely unmyelinated. Further transmission electron microscopy (TEM)-based analysis will be required to ascertain whether the MBP+ associated neuronal fibers have mature dense, myelin wrappings.

Given the changes found in myelination in adult SZ brains (Kubicki et al., 2007; Samartzis et al., 2014; Wheeler and Voineskos, 2014), these early changes in neuronal and oligodendrocytic development and myelination revealed in the organoid model support a delayed and abnormal development of mature myelinated neuronal systems and the SZ disconnection syndrome associated with white matter loss in the frontal and temporal lobes, as well as in the corpus callosum and internal capsule (Vanes et al., 2018).

### SZ and TNF Change Microstructure of the Organoid Cortex

The changes in neuronal cortical networks revealed by pan-Neu immunocytochemistry in SZ in an earlier study (Stachowiak et al., 2017) and the current study, and the changes in SZ+TNF, and relatively smaller changes in C+TNF organoids in the present study, were further illuminated by SEM. In all conditions that led to cortical underdevelopment (SZ, C+TNF, SZ+TNF), the paucity of the cell bodies was accompanied by increased spaces between cells that were “vacant” in C+TNF but became filled with the ECM fibrous material in SZ and in SZ+TNF organoids. This suggests that SZ promotes ECM development, consistent with the presence of ECM regulating genes within the SZ dysregulated transcriptome (Narla et al., 2018). Such changes may alter the mechanical properties (surface tension) of the developing cortex, which could further influence the development of the brain cortex (Kroenke and Bayly, 2018) and, potentially, lead to mild cerebral atrophy observed in SZ patients (Weinberger et al., 1979).

The cerebral organoid findings are consistent with MRI data of brains from patients with SZ that showed a specific decrease in both cortical thickness and complexity (sulci and gyri), along with enlarged ventricles. The areas that were the most heavily impacted were the temporal and frontal lobes. The authors also note that these changes may be rooted in the abnormal neural development that occurs *in utero* (Haukvik et al., 2013).

### Mechanisms of Cellular Changes—Role of INFS

**Figure 7:**
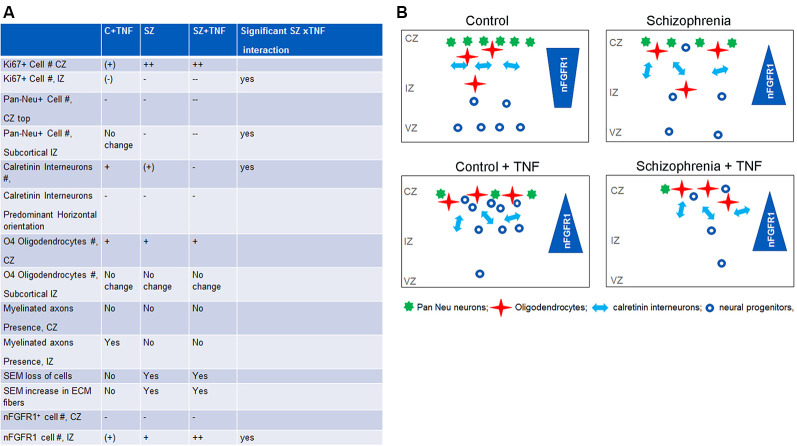
Comparisons of the effects of TNF, SZ, and their combined effect on the organoid development. **(A)** Significant increase: +; significant decrease: −; significant differences: +/++,–/−−; non-significant trend (+), (−). The effects of TNF were either similar or less pronounced than of SZ. In some cellular changes, the effects of SZ+TNF were bigger than the individual factors. In one case, density of calretinin interneurons, the interaction between TNF and SZ reversed their individual effects (increases) to a marked loss of interneurons by SZ+TNF. **(B)** The relationship of INFS and altered neuronogenesis and oligodendrogenesis by TNF and in SZ. During brain development, the nuclear location of nFGFR1, which programs neuronal development, gradually increases as the proliferating neural progenitor cells (NPC) progress through neuroblasts to differentiating neurons that form cortical layers. The diverse SZ-linked mutations that disturb developmental signals and an increased TNF promote premature nuclear FGFR1 location leading to increased formation of neurons already within the VZ and IZ and reduced subcortical oligodendrocytic development. However, when cells reach the organoid surface, nFGFR1 is turned off, possibly by increased ECM signals (Stachowiak et al., 2017). The loss of nFGFR1 reduces formation of dense cortical neuronal networks, while increasing numbers of short calretinin interneurons, diminishing their preferred horizontal orientation, and promoting oligodendrocytes. The TNF-induced changes in C organoids are similar, although generally less pronounced than those occurring in SZ. On the other hand, the combined effect of SZ and TNF are often more pronounced or even opposite (loss of calretinin interneurons) from their individual effects, likely reflecting INFS dysregulation of TNF receptor signaling. Our findings suggest that the combined effects of the SZ genomic dysregulations and maternal immune activation (MIA) may increase the risk and/or severity of SZ.

In the VZ and IZ of the control organoids, FGFR1 was predominantly cytoplasmic and became increasingly nuclear as the cells reached the CZ (also Stachowiak et al., 2017). This is consistent with the changing function of FGFR1, initially as the mitogenic plasma membrane receptor in the VZ and IZ, gradually to the differentiation programing nuclear protein in the IZ and CZ. The increased numbers of IZ cells with nFGFR1 in SZ+TNF, similar to the albeit pronounced trend in C+TNF and SZ, and the significantly enhanced by TNF in SZ organoids, indicated that both factors, disease and TNF, stimulate INFS in the IZ in a synergistic manner (Figure 7A).

The current investigation confirmed the loss of nFGFR1 in cortical cells of the SZ organoids (Stachowiak et al., 2017), and showed that, like in SZ, TNF disruption of cortical development (Figure 3) coincides with the loss of nFGFR1 expressing cells in the developing cortex. Thus, excess TNF appears to impair pan-developmental INFS, essential for cortical neuronal development.

The overactivation of INFS in the subcortical region and its inactivation in the cortex is thought to underlie the premature NPC development in subcortical loci and an impaired development in the organoid cortex, as shown in cultured NPC and organoids (Stachowiak et al., 2017; Chuye et al., 2018). The relationship of INFS and altered neuronogenesis and oligodendrogenesis by TNF and in SZ are summarized in Figure 7B.

In studies of SZ iPSC NPC, it was shown that dysregulation of INFS had opposite effects on the expression of genes involved in the generation of oligodendrocytes and neurons (Narla et al., 2017). nFGFR1 was shown to inhibit oligodendrogenic genes and activate neuronogenic genes. These findings can now be translated into the cellular data of the present organoid study. Increased subcortical nFGFR1 in disrupted conditions was accompanied by pan-Neu^+^ neuronal foci, but with fewer IZ cells expressing O7294. The opposite changes were found in the cortex, where upon with the loss of nFGFR1, pan-Neu^+^ cells were restricted from the cortex and Olig4^+^ cells were more abundant than in control (non-TNF exposed) organoids. These opposite changes in the distribution of oligodendrocytic and neuronal cells may reflect the effects of nFGFR1 on neuronal (stimulation) and glial/oligodendrocytic (inhibition) genes in developing NPC (Narla et al., 2017). The current *in-silico* analyses revealed direct nFGFR1 targeting of the promoters of these critical genes in human developing NPC, and its increase in SZ, as the genes became dysregulated. Hence, the changes in nFGFR1, as they occur in SZ and induced by TNF, may directly affect these critical genes and cell lineage developmental decisions.

The significant interaction of SZ and TNF in eliciting changes in neuronal and oligodendrocytic populations (Figure 7A) and in nFGFR1 indicated an increased sensitivity of the developing SZ brain to TNF, and thus, MIA. The analyses of the dysregulated genes in SZ NPC enlightens changes in the expression of TNF receptors and signaling pathway genes as a potential mechanism for the increased TNF sensitivity in SZ NPC. In all four SZ patients, a multifold upregulation of the TNF receptor-II genes TNFRSF1B and TNFRSF10B, dubbed the “death receptor,” as well as TNF receptor 1 signaling genes PAK1 and PAK7, was found. The serine/threonine-protein kinases, PAK1 and PAK7, which influence a wide variety of cellular processes, including directional motility and growth and are overexpressed in many diseases, showed multi-fold upregulation in SZ cells (GSE92874; Narla et al., 2017).

TNF is a strong candidate gene for SZ. Serum levels of TNF were significantly increased in SZ, associated with more severe symptoms of SZ and with a higher risk of developing SZ (Suchanek-Raif et al., 2018). The mining of the existing FGFR1 ChiPseq data from our laboratory revealed that nFGFR1 direct binding to TNF receptor genes, as well as to the component genes of their receptor pathways, was increased in SZ (Supplementary Figure S11). Through these changes, the developing SZ brain with inherited genome dysregulations may suffer increased vulnerability to TNF, which is neurotoxic when in excess. This further lends support to the proposal that MIA could be a significant contribution for genetically at-risk fetuses for the expression of SZ.

Since a single representative SZ iPSC line was used for the extensive quantitative-computational anatomical analyses in the present studies, whether the supersensitivity to TNF similarly extends to the other SZ lines, as well as the extent to which individual genotypes may affect the response to TNF and the potential treatments are the subject of ongoing studies. The genomic changes in TNF receptors and their signaling pathway genes found in multiple SZ patients’ iPSCs (Narla et al., 2017) are consistent with the SZ patient-derived organoid supersensitivity to TNF.

In conclusion, cerebral organoid and genomic-based models offer plausible cellular and molecular mechanisms for TNF-dependent neurodevelopmental pathology of SZ and its induction by MIA.

## Data Availability Statement

The RNAseq datasets for this study can be found on with the accession code: GSE92874. The ChiPseq datasets analyzed in the present study can be accessed at GSE92873.

## Author Contributions

CB: doctoral student, dissertation project, performed the major portion of experiments, analyzed the data, and assisted with writing the manuscript. HP: Master’s student thesis project, performed some of the experiments and assisted with manuscript writing and editing. ML: assisted with experiments and contributed to manuscript preparation and editing. SD and DF: performed some of the experiments and contributed to manuscript preparation and editing. TI: contributed to experimental design, data interpretation, and contributed to manuscript writing and editing. ES: contributed to experimental design, data analysis, and writing of the manuscript. MS: overall project design, oversee all aspects of project, data analysis and interpretation, and wrote the manuscript.

## Conflict of Interest

The authors declare that the research was conducted in the absence of any commercial or financial relationships that could be construed as a potential conflict of interest.

## Abbreviations

C: control
SZ: Schizophrenia
MIA: maternal immune activation
TNF-α: Tumor Necrosis Factor-α
ESC: embryonic stem cells
iPSC: induced pluripotent stem cell
NPC: neural progenitor cells
VZ: ventricular zone
CZ: cortical zone
IZ: intermediate zone
FGFR: fibroblast growth factor receptor
INFS: integrative nuclear (n) FGFR1 signaling
CBP: CREB binding protein
ROI: regions of interest
SEM: scanning electron microscopy
TBR1: transcription factor T-Box Brain 1
MBP: myelin basic protein
ECM: extracellular matrix.
