# Supplementary material for: Immune Factor, TNFα, Disrupts Human Brain Organoid Development Similar to Schizophrenia—Schizophrenia Increases Developmental Vulnerability to TNFα
Source: Front Cell Neurosci. 2020 Aug 28;14:233. doi: 10.3389/fncel.2020.00233 (PMC7484483; doi:10.3389/fncel.2020.00233)

Supplementary Table 1 – antibodies used for immunostaining.

Primary Antibodies:

Antibody	Rabbit	Mouse	Isotype	Dilution	Company	Catalog No
Caspase 3	+		IgG	1:150	Abcam	ab44976
Calretinin	+		IgG	1:500	Abcam	ab16694
GFAP	+		IgG	1:1000	Millipore	ab5804
Ki67	+		IgG	1:500	Abcam	ab15580
Myelin basic protein	+		IgG	1:100	Millipore	ab9348
O4		+	IgM	1:330	Neuromics	MO15002
PanNeu		+	IgG1	1:250	Millipore	MAB2300
FGFR1	+		IgG	1:100	Abcam	ab16694
TBR1	+		IgG	1:400	Abcam	ab31940

Secondary Antibodies:

Antibody	Rabbit	Mouse	Isotype	Dilution	Company	
Alexa Fluor 488		+	IgM	1:1000	Abcam	ab150121
Alexa Fluor 488	+		IgG	1:1500	Invitrogen	A11070
Alexa Fluor 568	+		IgG	1:1000	Life Technologies	A10520
Alexa Fluor 488		+	IgG	1:1000	Abcam	ab150117
oat Anti-Mouse IgG H&L (Cy3)		+	IgG	1:500	Abcam	ab97035

Supplementary Figure S1. GFAP immunostaining (green), DAPI (blue) staining – tile scanning. (**a**) Control HUES8 organoid: GFAP$^+$ radial glia outline the VZ of the rosettes; * marks exemplary rosette. (**b**) TNF (1 ng/ml) exposed HUES8 organoid - clusters of GFAP$^+$ radial glia were dispersed throughout the organoids.

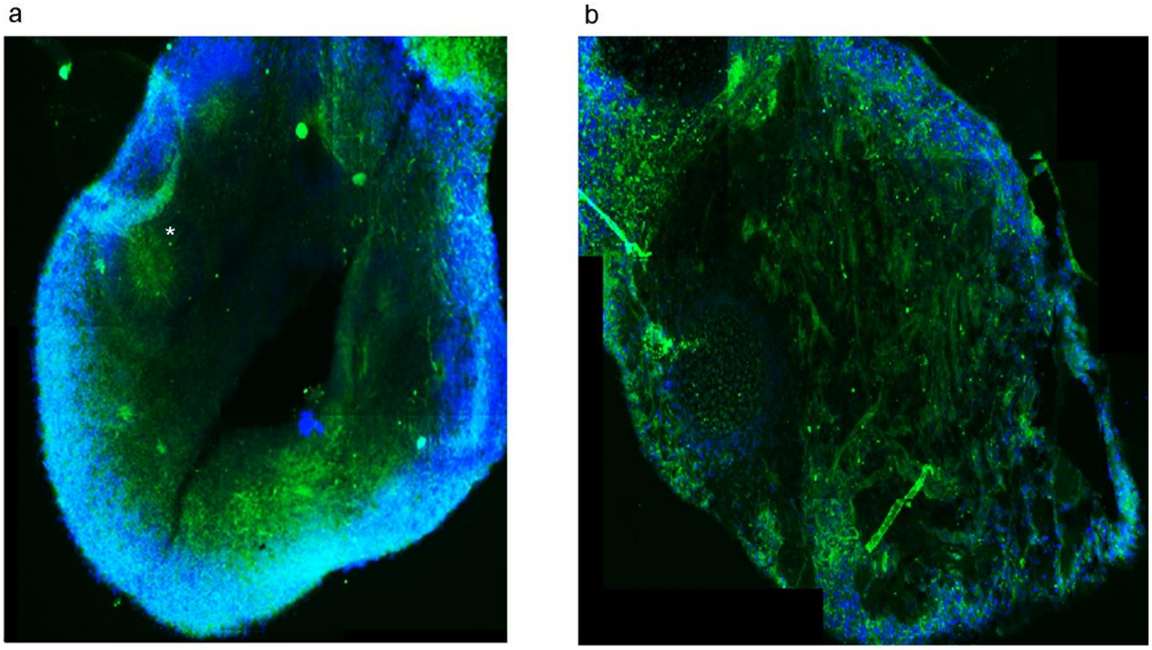

Supplementary Figure S2. Apoptosis in HUES8 organoids at 5 weeks. Images show few Caspase 3 immunostained cells in HUES8 control organoids (a) and increases in occurrence of Caspase 3^{+} cells in 1 ng/ml (b) and in 10 ng/ml TNF (c) exposed HUES8 organoids. Enlarged images at right show: (c1) Green (Caspase 3) and blue (DAPI) channels, (c2) only blue channel to illustrate nuclear localization of Caspase-3 signal**.**

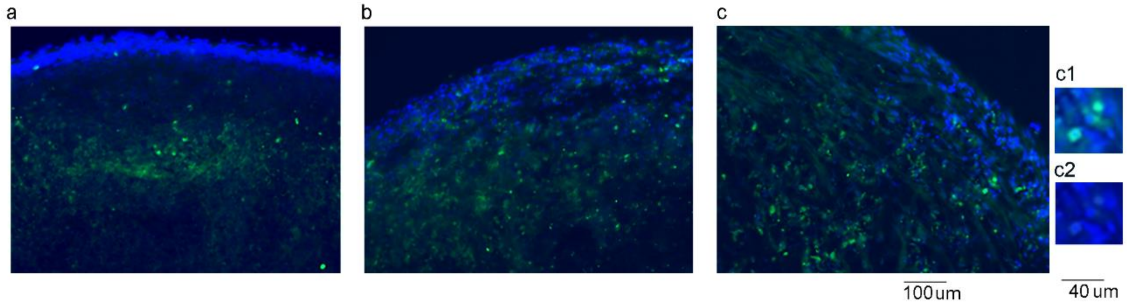

Supplementary Figure S3. Representative images of iPSC organoids at 5 weeks: (**a**) C, (**b**) SZ, (**c**) C+TNF, (**d**) SZ+TNF (50 pg/ml).

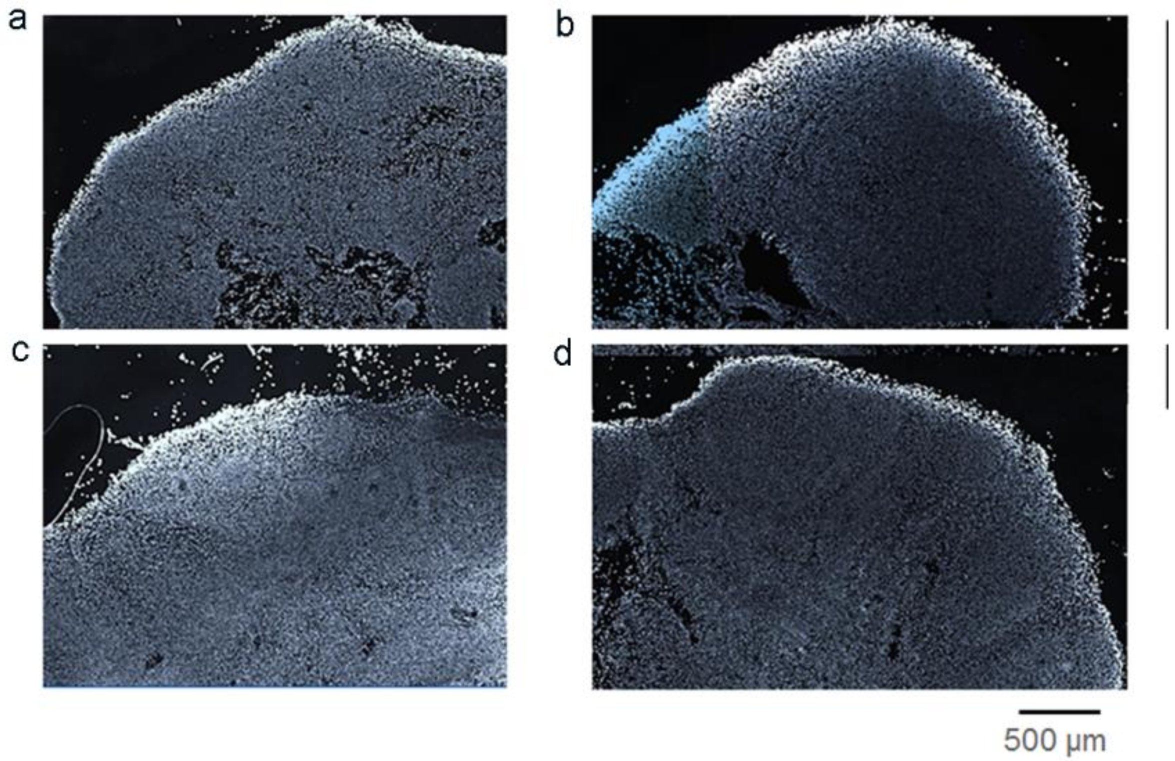

Supplementary Figure S4. Examples of iPSC organoids at 5 weeks; Pan-Neu (green) and DAPI (blue) staining, tile scanning. Representative images of organoids are shown: (**a**) control (C; non-TNF exposed), (**b**) C+TNF, (**c**) schizophrenia (SZ; non-TNF exposed), and (**d**) SZ+TNF (TNF 250 pg/ml).

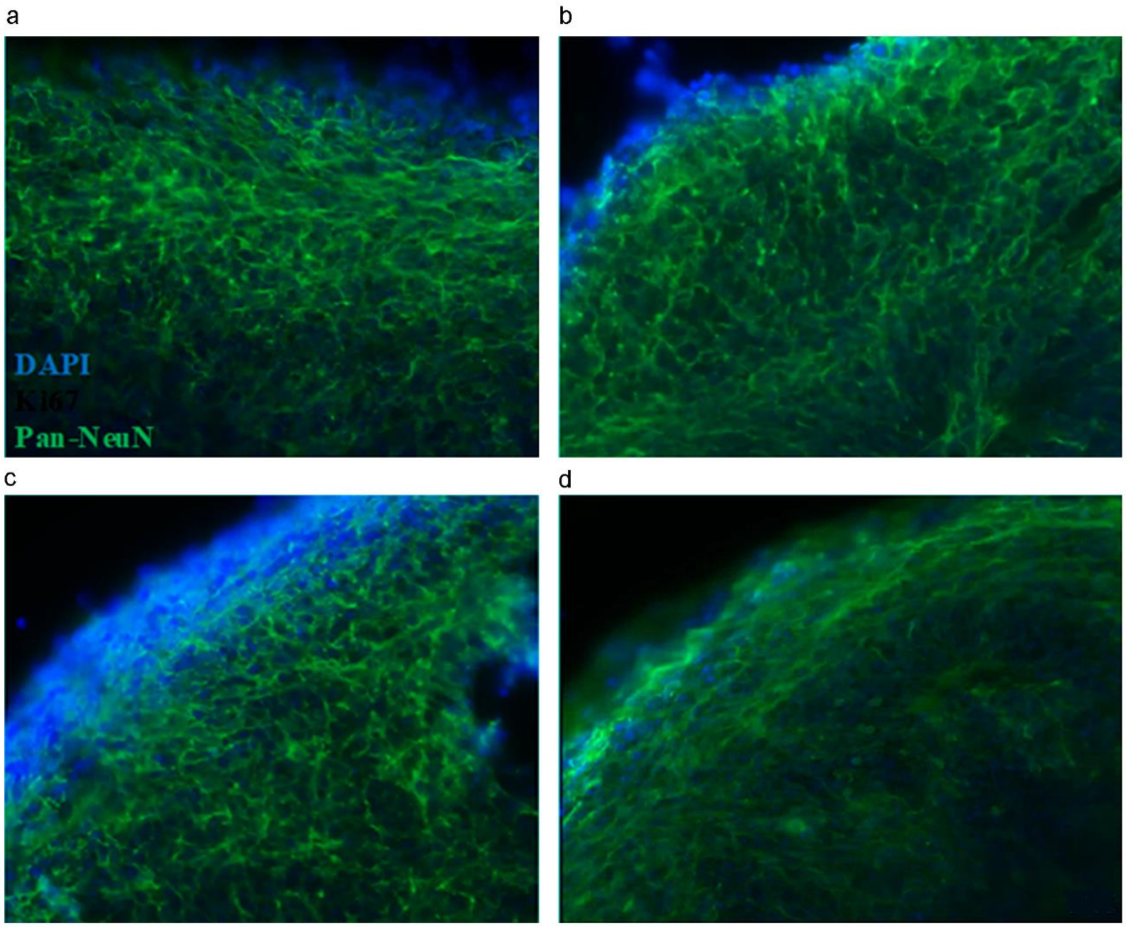

Supplementary Figure S5. TBR1 expression of neuroblasts/early pioneer neurons affected in SZ and TNF exposed organoids at 5 weeks. Images show immunostained TBR1 (red) and DAPI (blue) stained nuclei: (**a**) C, (**b**) SZ, (**c**) C+TNF, (**d**) SZ+TNF (50 pg/ml). Note high levels of expression in CZ and IZ of C organoid, and reduced expression of TBR1 in C+TNF, SZ, and SZ+TNF organoids.

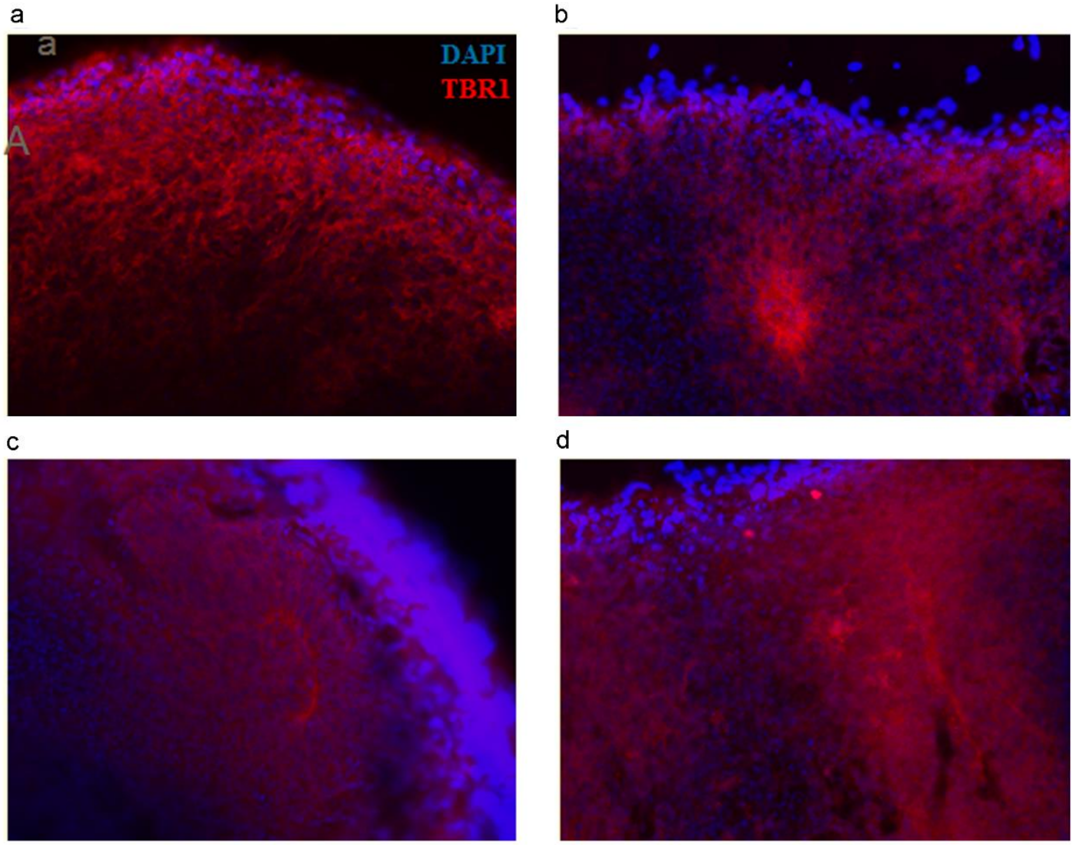

Supplementary Figure S6. Myelination of Pan-Neu neurons at 5 weeks. Organoid sections were co-immunostained for Myelin Basic Protein (red) and Pan-Neu (green) and stained with DAPI (blue). Organoids: (**a**) C, (**b**) SZ, (**c**) C+TNF, (**d**) SZ+TNF (50 pg TNF). In C organoids (**a**) Myelin Basic Protein and Pan-Neu staining were both detected in CZ and IZ, indicating the presence of myelinated neurons. The IZ staining was reduced in the C+TNF group. In the SZ and SZ+TNF organoids, the myelin staining was greatly diminished in the CZ and IZ, and the IZ neurons showed no myelination.

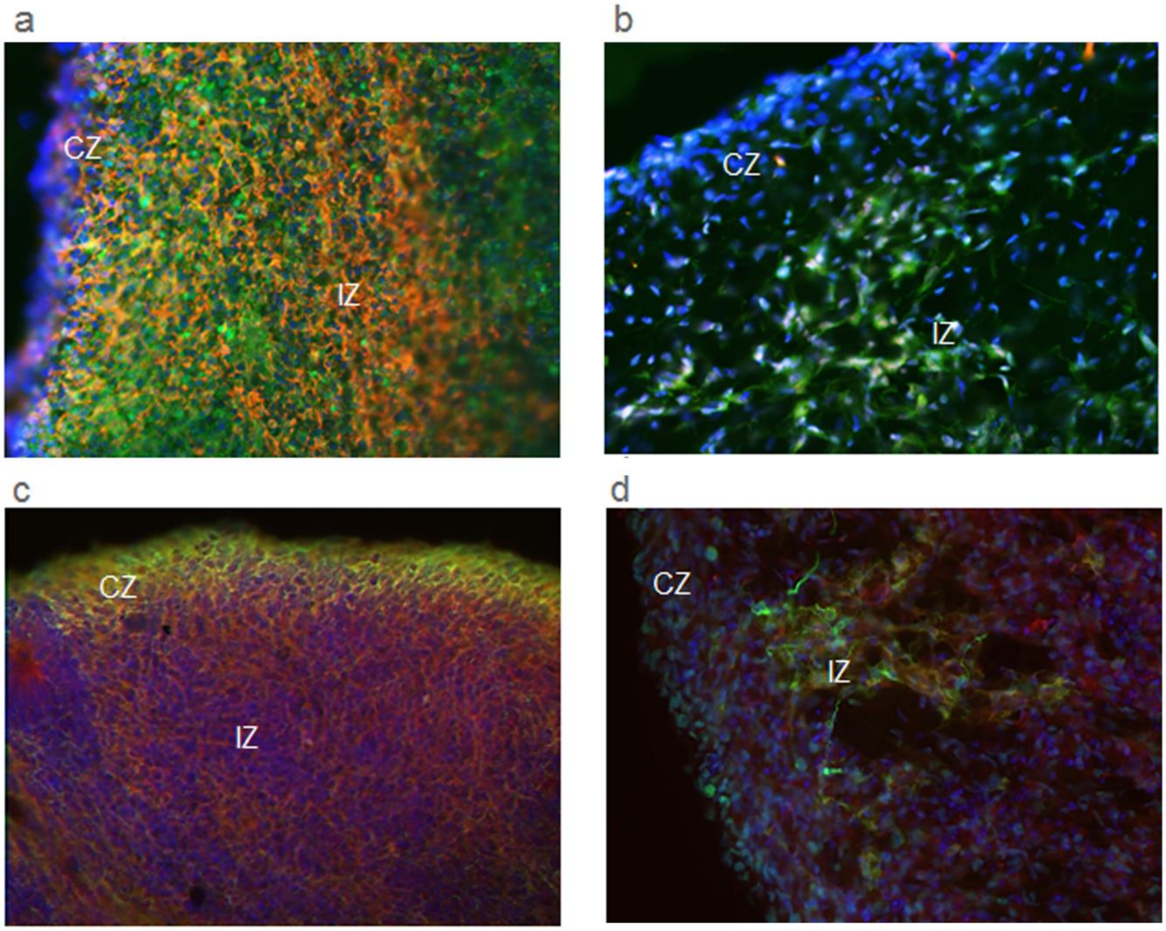

Supplementary Figure S7. nFGFR1 targets promoters of diverse neuronal genes. Examples of nFGFR1 binding to promoters of the neurogenic genes WNT7B and DISC1, and to neuronal light, medium and heavy neurofilament (NEFL, NEFM, NEFH) genes. Gene browser analysis performed on FGFR1 ChiPseq data sets (GSE92873) (3). Note differences in gene promoter targeting in control and schizophrenia NPC.

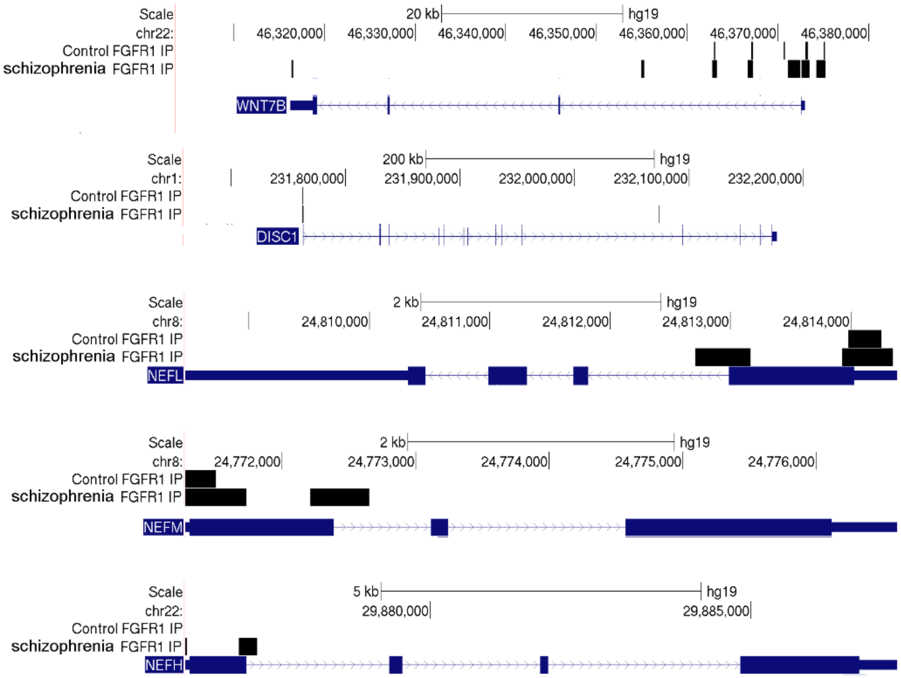

Supplementary Figure S8. Examples of nFGFR1 binding to the promoters of oligodendrogenic genes: Olig2 and SOX9. Gene browser analysis performed on FGFR1 ChiPseq data sets (Narla et al.). Note differences in gene promoter targeting in control and schizophrenia NPC. Examples of nFGFR1 non-binding gene Olig 1 is also shown. Gene browser analysis performed on FGFR1 ChiPseq data sets (GSE92873) (3).

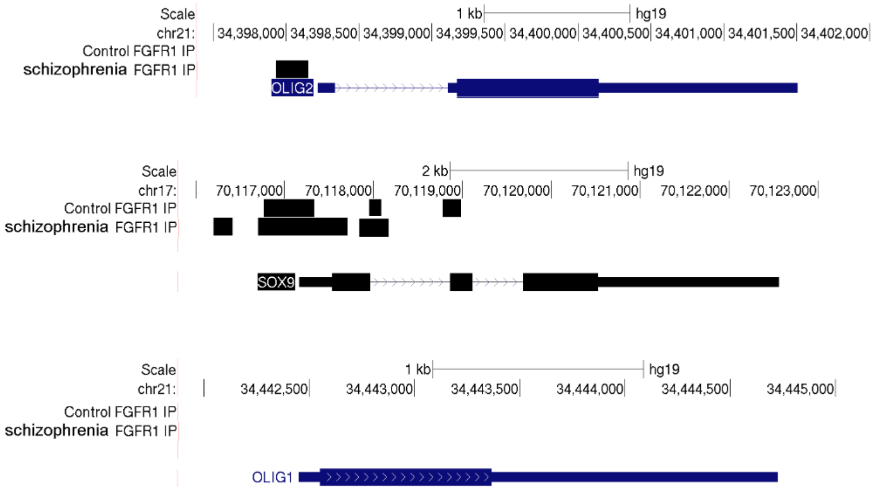

Supplementary Figure S9. nFGFR1 targets promoters of diverse ECM-related genes. Gene browser analysis performed on FGFR1 ChiPseq data sets (GSE92873) (3). Note differences in gene promoter targeting in control and schizophrenia NPC.

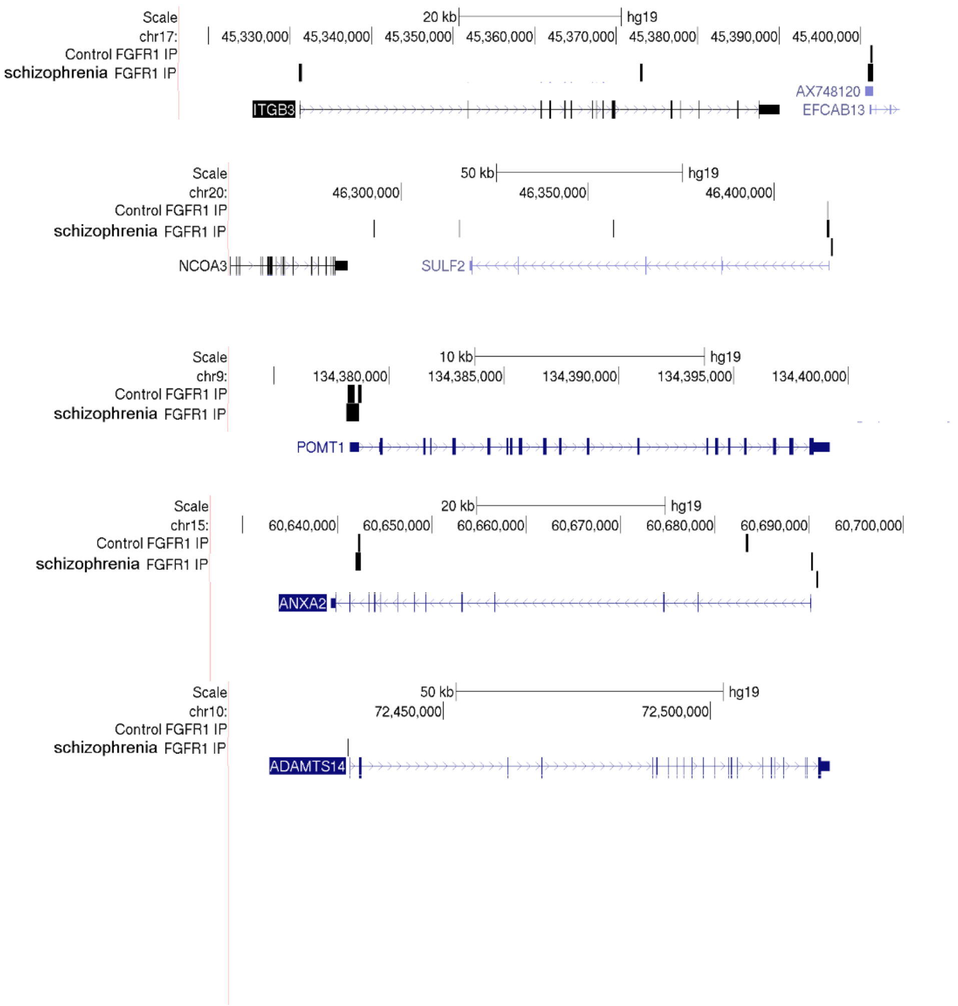

Supplementary Figure S10. nFGFR1 targets promoters of diverse cell migration genes. Gene browser analysis performed on FGFR1 ChiPseq data sets (GSE92873) (3).

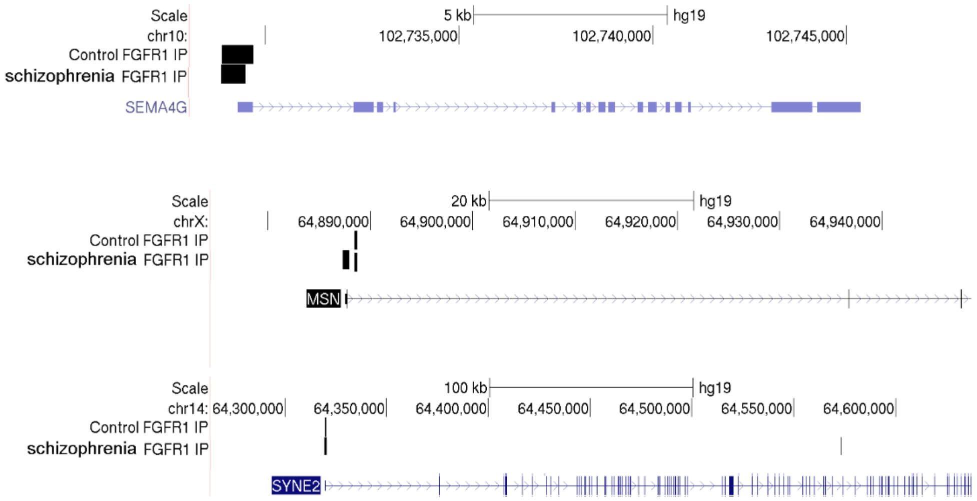

Supplementary Figure S11. TNF and nFGFR1 binding: nFGFR1 binds with a number of TNF receptor genes and to their signaling protein genes. TNFRSF10B is referred to as a "death receptor", While TNFRSF1B is referred to as TNFR2 (neuroprotective Supports survival). Note the increased nFGFR1 binding to the promoter regions of TNFRSF1B; PAK1 and PAK7 genes and its altered pattern at the promoter of TNFRSF10B in SZ NPC. Expression of all of these genes was increased several-fold in NPC of SZ IPSC compared to control NPC: Log2-fold: PAK1-3.3, PAK7-2.2 UP, TNFR1F -1.5; TNRFSF1B- 5.5. Gene browser analysis performed on FGFR1 ChiPseq data sets (GSE92873) (3).

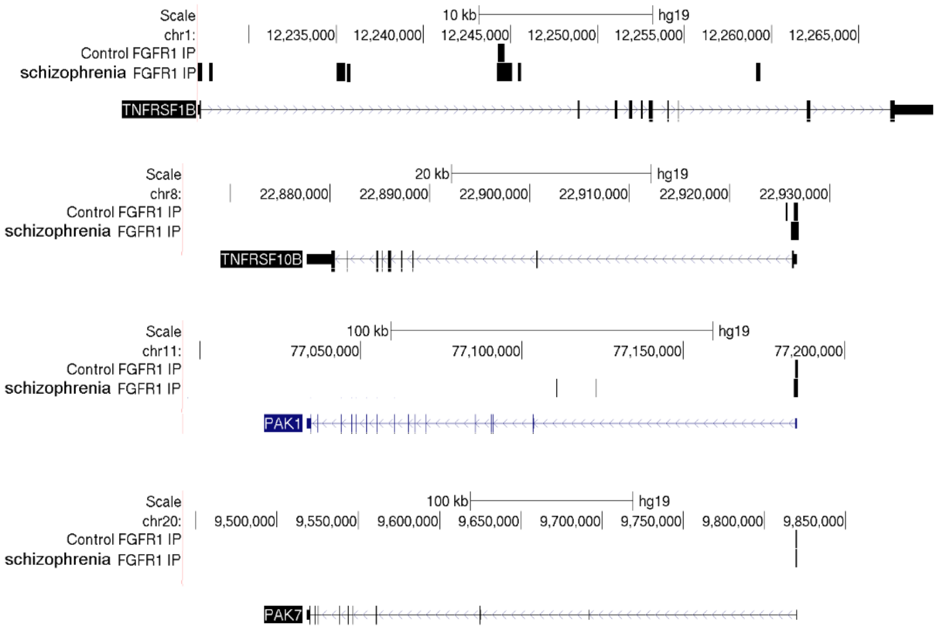

Supplement: Supplementary file 1 [file Data_Sheet_1.pdf]
